# Opportunistic detection of *Fusobacterium nucleatum* as a marker for the early gut microbial dysbiosis

**DOI:** 10.1186/s12866-020-01887-4

**Published:** 2020-07-13

**Authors:** Ji-Won Huh, Tae-Young Roh

**Affiliations:** 1 Division of Integrative Biosciences and Biotechnology, Pohang University of Science and Technology (POSTECH), Pohang, 37673 Republic of Korea; 2 Department of Life Sciences, Pohang University of Science and Technology (POSTECH), Pohang, 37673 Republic of Korea; 3 SysGenLab Inc, Pohang, 37673 Republic of Korea

**Keywords:** *Fusobacterium nucleatum*, Inflammatory bowel diseases (IBD), Colorectal cancer (CRC), Opportunistic detection, Microbial experience, Integrative human microbiome project (iHMP)

## Abstract

**Background:**

The essential roles of gut microbiome have been emphasized in modulating human health and disease. *Fusobacterium nucleatum* (*F. nucleatum*), an obligate Gram-negative microorganism residing in oral cavity, gastrointestinal tract and elsewhere, has been recently considered as a potential oncobacterium associated with human cancers. However, the consequence of its enrichment was not extensively explored in terms of microbial homeostasis and stability at the early stage of disease development.

**Result:**

Our analysis on longitudinal metagenomic data generated by the Integrative Human Microbiome Project (iHMP) showed that *F. nucleatum* was frequently found in inflammatory bowel diseases (IBD) subjects with reduced microbial diversity. Using non-parametric logarithmic linear discriminant analysis (LDA) effect size (LEfSe) algorithm, 12 IBD- and 14 non-IBD-specific bacterial species were identified in the fecal metagenome and the IBD-specific ones were over-represented in the *F. nucleatum*-experienced subjects during long-term surveillance. In addition, *F. nucleatum* experience severely abrogated intra-personal stability of microbiome in IBD patients and induced highly variable gut microbiome between subjects. From the longitudinal comparison between microbial distributions prior and posterior to *F. nucleatum* detection, 41 species could be proposed as indicative “classifiers” for dysbiotic gut state. By multiple logistic regression models established on these classifiers, the high probability of experiencing *F. nucleatum* was significantly correlated with decreased alpha-diversity and increased number of biomarker species for IBD and colorectal cancer (CRC). Finally, microbial clustering confirmed that biomarker species for IBD and non-IBD conditions as well as CRC signature markers were well distinguishable and could be utilized for explaining gut symbiosis and dysbiosis.

**Conclusion:**

*F. nucleatum* opportunistically appeared under early dysbiotic condition in gut, and discriminative classifier species associated with *F. nucleatum* were successfully applied to predict microbial alterations in both IBD and non-IBD conditions. Our prediction model and microbial classifier biomarkers for estimating gut dysbiosis should provide a novel aspect of microbial homeostasis/dynamics and useful information on non-invasive biomarker screening.

## Background

The microbial communities in the gastrointestinal tract play pivotal roles in maintaining many biological functions such as food digestion, metabolism, and immunological regulations as well as developing diseases like ulcers, bowel perforation, inflammatory bowel diseases, irritable bowel syndrome, other inflammatory conditions, metabolic syndromes, and even cancers.

*F. nucleatum* was initially identified as a non-motile obligate anaerobe commonly residing on the tooth surface of healthy individuals and bridging bacterial species to form dental plaque [1, 2]. Many researches have reported that *F. nucleatum* is ectopically colonized in distal organs and associated with several disorders such as adverse pregnancy outcomes, IBD, Lemierre’s syndrome, cardiovascular diseases, atherosclerosis, Alzheimer’s disease, and cancers [3–8]. IBD refers to as chronic conditions describing a group of inflammatory disorders in intestines. Patients with IBD tend to show a high level of *F. nucleatum* in the colon and are at significantly high risk of CRC. It has been demonstrated that *F. nucleatum* is related with and promotes the growth of CRC [9–17].

CRC is the fourth most incident cancer in the world. The rates of CRC incidence and mortality are still rising in developing countries and in relatively young people in the United State [18, 19]. Chronic inflammation at large intestine is a significant risk factor of CRC [20, 21]. The patients with IBD are six times more likely to develop CRC when compared with control group. CRC accounts for one out of seven deaths in IBD patients [22]. Furthermore, the incidence of CRC after a negative colonoscopy is three times higher in IBD patients than in healthy controls, indicating that chronic inflammation facilitates colorectal tumor promotion [23]. For early detection of CRC, endoscopic surveillance is usually recommended but people are reluctant to the uncomfortable test, resulting in late diagnosis and poor prognosis of CRC. Thus, there is a realistic need for development of non-invasive and potent biomarkers for the early CRC diagnosis [24]. Despite the differential enrichment of *F. nucleatum* in CRC tissues, the effectiveness of fecal *F. nucleatum* as a potential non-invasive biomarker is still underestimated due to its rare appearance in stool [25–32].

The iHMP released extensive longitudinal datasets of disease-specific cohorts to understand the interaction between the microbiome and host using multi-omics technologies. Among them, there are shotgun metagenomic sequencing data from IBD fecal samples of 130 people over 1 year [33]. Recently, a multi-institutional group reported the comprehensive profiling of overall metagenome using IBD databases from iHMP but the establishment of cancer-associated microbiome in IBD patients has not been investigated [34].

Here we examined whether *F. nucleatum* and its associated pathobionts might be promising biomarker species reflecting dysbiotic environment by analyzing the longitudinal metagenomic data and predicted if the occurrence of *F. nucleatum* could play a function as an indicator of disease condition.

## Results

### Metagenomic profiling of IBD or non-IBD participants

**Fig. 1:**
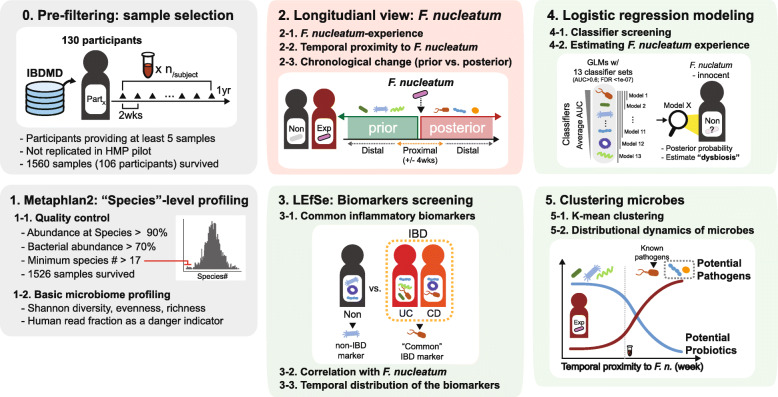
Schematic diagram of metagenome analysis. Longitudinal metagenome data from IBDMD were filtered by indicated criteria, and basic characteristics of microbiome were profiled. Based on longitudinal experience of *F. nucleatum* and temporal distribution toward *F. nucleatum*-detected samples, microbial characteristics was compared. Using LEfSe algorithms, microbial biomarkers of non-IBD or common IBD condition were screened, and correlation of the marker species with *F. nucleatum* was assessed. After identifying classifier microbes, which significantly differentiate *F. nucleatum*-observed point, probability of experiencing *F. nucleatum* was estimated in *F. nucleatum*-innocent subjects using multiple logistic regression models. At last, microbes were classified into 9 clusters according to five longitudinal features associated with inflammatory conditions and *F. nucleatum* experience. Particular clusters contained a significant number of disease-associated marker species or well-known probiotics

As summarized in Fig. 1, the overall metagenomic analysis includes filtering, profiling, longitudinal dissection, biomarker screening, modeling, and microbial dynamics test. The fecal metagenome dataset used in this study was downloaded from the Inflammatory Bowel Disease Multi-'Omics Database (IBDMD) of iHMP, which were longitudinally generated from 130 participants (103 IBD and 27 non-IBD subjects).

As described in Methods, the data quality was tested and metagenomic samples from valid subjects satisfying selection conditions were considered for further analysis (Additional file 1: Table S1). Microbial taxonomy was assessed at the species level using MetaPhlAn2, and the quality of the compositional data were controlled according to the three specific conditions mentioned in Methods (Additional file 2: Table S2) [35]. The number of filtered samples was 1526 samples from 106 participants (80 IBD and 26 non-IBD), and the metadata of participants such as sex, age, and collection days were comparable between IBD patients and non-IBD subjects (Additional file 3: Table S3). The global distribution did not show distinct tendency to sex, IBD-activity, subject, and data generation sites (Additional file 4: Figure S1).

**Fig. 2:**
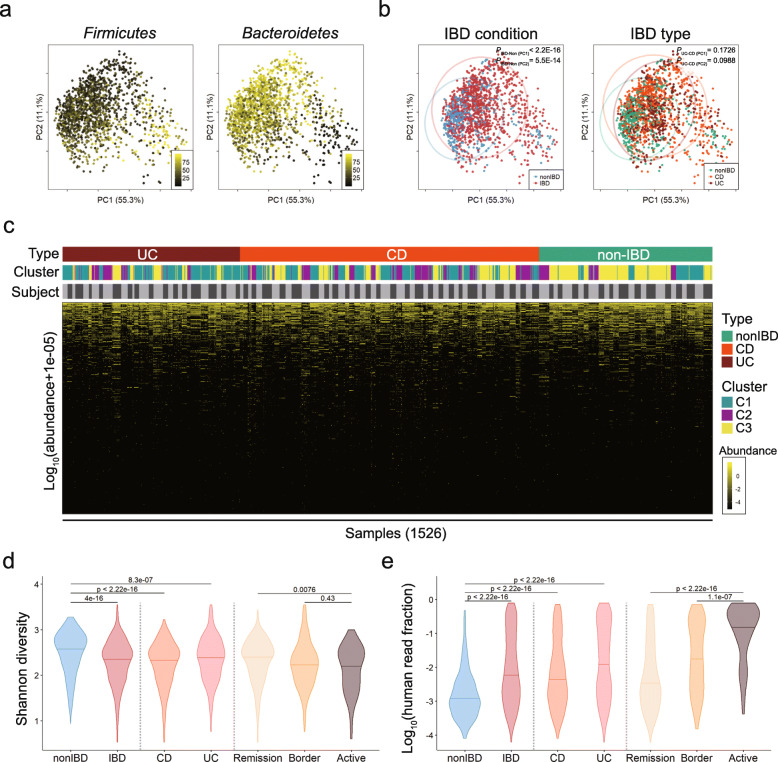
Characteristics of IBD and non-IBD microbiome data. **a** Reciprocal patterns of *Firmicutes* and *Bacteroidetes* on principal coordinate analysis (PCoA) plot. **b** Distribution of IBD and non-IBD (ulcerative colitis (UC), and Crohn’s disease (CD) samples on PCoA plot. *P*-values indicate a significance in pairwise comparison between two groups against principal coordinates. **c** Logarithmic abundance heatmap of 1526 samples. Pseudo-abundance (1e-05) was added to avoid infinite value. Samples were ordered by participant and visit number information. **d** Shannon diversity of samples. According to disease severity score, UC and CD samples were categorized into three stages (remission, border, and active). **e** Logarithmic human read fraction of samples

Consistent with the previous reports, two major phyla in human gut, *Firmicutes* and *Bacteroidetes* showed a complementary distribution in the plot of principal coordinate analysis (PCoA) (Fig. 2a) [36]. The microbiomes of IBD and non-IBD subjects were generally distinguishable. Samples of non-IBD subjects were mainly localized in a left-lower quadrant and ones of IBD patients were more widely distributed along PC1 axis (probability value of IBD vs. non-IBD, *P*_IBD-Non (PC1)_ < 2.2e-16, Fig. 2b). The representative subtypes of IBD, ulcerative colitis (UC) and Crohn’s diseases (CD), were not significantly segregated by PC1 and PC2 axes (*p*-values of UC vs. CD, *P*_UC-CD (PC1)_ = 0.1726, *P*_UC-CD (PC2)_ = 0.0988), implying that the two idiopathic inflammatory disorders share similar microbial community (Fig. 2b). Overall microbiome seemed to be distinct by subjects and largely stable over time (Fig. 2c). As grouped by K-means clustering, most of non-IBD samples belonged to cluster C3, suggesting that microbiome from non-IBD subjects should be relatively convergent relative to those from UC or CD (Odd Ratio (OR)_nonIBD-C3_ = 4.42, OR_UC-C3_ = 2.30, OR_CD-C2_ = 2.15).

Multiple alpha diversity indices like Shannon diversity, Pielou’s evenness, and richness (the number of observed species per sample) were lower in samples with IBD than in those without IBD as expected. There were no significant differences in alpha diversity indices between CD and UC, but severe inflammation lowered Shannon diversity and richness (Fig. 2d, Additional file 5: Figure S2a, b).

In addition, the fraction of human reads sequenced together with gut metagenomic data was high in IBD rather than in non-IBD and highest in active stage of IBD among three stages of IBD, which means that a leakage of host genome into gut lumen might mirror the severity of disorders in gut (Fig. 2e). Accordingly, the human sequence read fraction was positively correlated with diseases severity scores such as simple clinical colitis activity index (SCCAI) for UC and Harvey-Bradshaw index (HBI) for CD (Additional file 5: Figure S2c, d).

### Detection of *F. nucleatum* and its longitudinal dissection

**Fig. 3:**
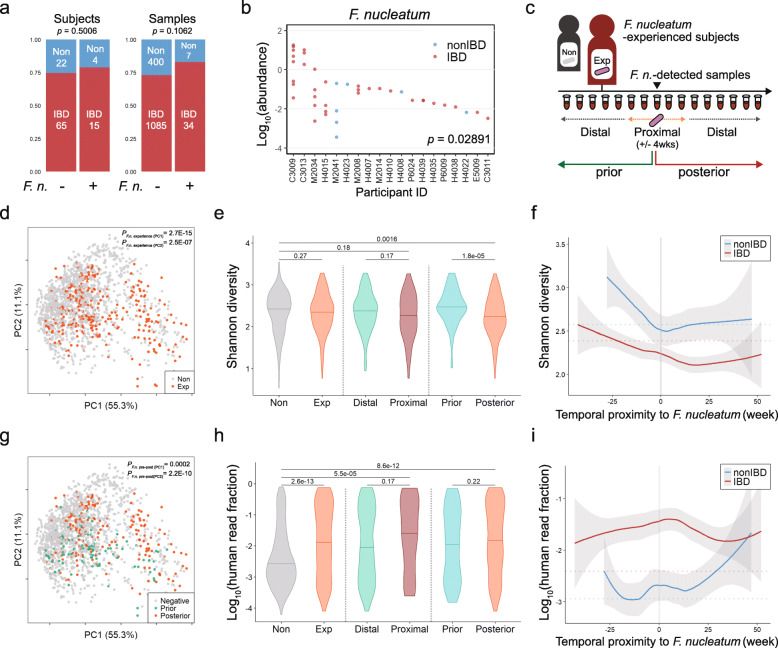
Transient colonization of *F. nucleatum* is a sign of intestinal disturbance. **a** IBD and non-IBD frequency by *F. nucleatum* observation. **b** Logarithmic abundance of *F. nucleatum* upon observation by subjects. Wilcoxon rank-sum test was conducted. **c** Sample classification by *F. nucleatum* experience, temporal proximity, and directionality. **d** Distribution of samples collected from *F. nucleatum*-experienced subjects. **e** Shannon diversity by *F. nucleatum*-oriented classification. **f** Shannon diversity of samples from *F. nucleatum*-experienced subjects based on temporal proximity to *F. nucleatum*-detected point. **g** Distribution of samples collected before or after the *F. nucleatum*-detected samples. **h** Logarithmic human read fraction of samples by *F. nucleatum*-oriented classification. **i** Logarithmic human read fraction of samples from *F. nucleatum*-experienced subjects based on temporal proximity to *F. nucleatum*-detected point

*F. nucleatum* is rarely found in gut microbiome. Among 1526 fecal samples from 106 participants, *F. nucleatum* occurred 41 times in 19 subjects (15 IBD and 4 non-IBD). The ratio of IBD to non-IBD subjects was not significantly different in *F. nucleatum*-detected subjects (Fisher’s one-sided test, *p*-value 0.4757), but the ratio of IBD to non-IBD samples had marginal preference to chronic inflammation due to the recurrent observation of *F. nucleatum* in IBD patients (OR = 1.79, Fisher’s one-sided *P*_detect_ = 0.1062) (Fig. 3a). However, *F. nucleatum* was relatively abundant within samples of IBD patients experiencing *F. nucleatum* (Wilcoxon test *P*_detect_ = 0.02891) (Fig. 3b).

Even though the low detection frequency of *F. nucleatum* is not appropriate for early diagnosis of disease state, it would be a constructive approach of overcoming this constraint to examine the longitudinal metagenomes before and after detection of a certain species along with co-occurring species. Firstly, we tested whether the detection frequency and abundance were consistent in 44 duplicated samples that were sequenced in both Human microbiome project (HMP) and HMP pilot study individually. Microbial abundance and the detection frequency are positively correlated and the recovery rate is usually high for abundant species. As expected, highly abundant species were found in duplicates but less abundant ones with abundance below 0.01%, were not. About one-fourth of total species appeared only in one sample of a given duplicated pair. *F. nucleatum* was a relatively rare microbe observed only 4 times in three duplicates and its recovery rate was only 33.3% (Additional file 6: Figure S3). To overcome the limitation of snapshot-based approach, the samples collected from each subject over 1 year were arranged in chronological order relative to the detection point of *F. nucleatum* (Fig. 3c). The subjects were categorized into *F. nucleatum*-experienced or –innocent (non-experienced) groups, and the samples from *F. nucleatum*-experienced subjects were sub-divided into prior or posterior group as well as proximal or distal group to the detection point of *F. nucleatum*. The samples of *F. nucleatum*-experienced subjects were highly dispersed in PCoA plot (Fig. 3d, g). Experiencing *F. nucleatum* led to lowering Shannon diversity and Pielou’s evenness. Particularly, the samples either proximal or posterior to *F. nucleatum* detection exhibited decreased alpha diversity and increased human read fraction (Fig. 3e, h, i, Additional file 5: Figure S2e, f). Longitudinal tracking of *F. nucleatum*-experienced subjects revealed that the microbial diversity was decreased in non-IBD subjects as well as in IBD patients (Fig. 3f). These results imply that *F. nucleatum* might appear under gut microbiome perturbation toward a low microbial diversity.

### Identification of biomarkers in IBD/non-IBD and their correlation with *F. nucleatum*

**Fig. 4:**
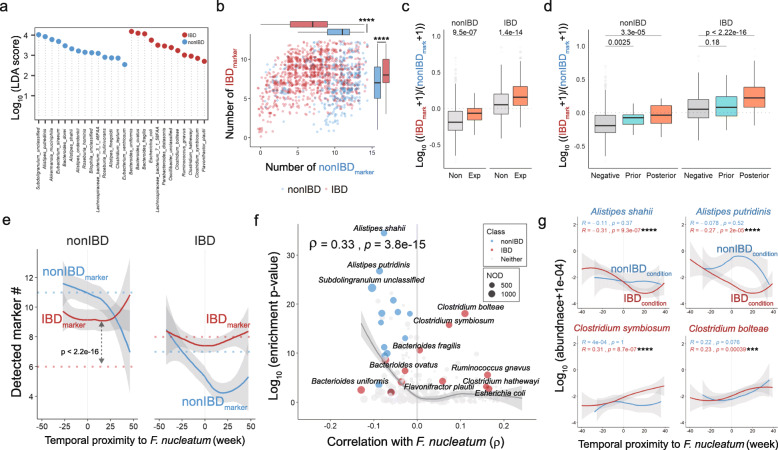
Microbial biomarkers for inflammatory conditions highly correlated with *F. nucleatum*. **a** Screening non-IBD or IBD marker species by LEfSe algorithm. Y-axis indicated logarithmic linear discriminant analysis (LDA) score. **b** Number of detected marker species per sample by inflammatory condition. **c** Logarithmic detection ratio of IBD/non-IBD marker species detected depending on the experience of *F. nucleatum*. Pseudo-count 1 was added to denominator and numerator to avoid infinite value. **d** Logarithmic detection ratio of IBD/non-IBD marker species by temporal distribution toward *F. nucleatum* detection. **e** Distribution of IBD and non-IBD marker species along temporal proximity to *F. nucleatum* observation in *F. nucleatum*-experienced subjects (solid lines). Dotted lines indicate the median number of detected marker species in *F. nucleatum*-innocent subjects (red: IBD biomarkers, blue: non-IBD biomarker). Gray arrow shows the comparison of the number of detected IBD biomarker in F. nucleatum-experienced and -innocent subjects. **f** Relationship between Spearman correlation coefficients of biomarker species with *F. nucleatum* and differential enrichment *p*-value of the microbes in IBD or non-IBD condition. Circle size denotes the number of detection (NOD) of the microbe across whole samples. **g** Logarithmic abundance of four representative IBD and non-IBD marker species along the temporal axis centered at *F. nucleatum*-detection. Blue line indicates non-IBD and red line, IBD. Font color for microbes indicates marker classes (blue for non-IBD; dark red for IBD). * indicates *p*-value < 0.05, ** *p* < 0.01, *** *p* < 0.001, **** *p* < 0.0001

In order to clarify whether *F. nucleatum* was truly associated with inflammatory environment, we tried to screen biomarker species for IBD and non-IBD conditions. Using a non-parametric Linear discriminant analysis Effect Size (LEfSe) algorithm, 12 IBD- and 14 non-IBD-specific biomarkers were selected at the species level (Fig. 4a, Additional file 7: Table S4). As expected, these markers were differentially enriched in either IBD or non-IBD samples (Fig. 4b). The ratio of IBD markers to non-IBD markers was significantly increased in *F. nucleatum*-experienced subjects, suggesting that more IBD-specific biomarkers were associated with detection of *F. nucleatum* whether or not IBD was developed (Fig. 4c). The prevalence of IBD markers over non-IBD markers was also distinct in samples posterior to *F. nucleatum*-detection (Fig. 4d).

As shown in Fig. 4e, the number of IBD-specific biomarkers is an indicator for *F. nucleatum* occurrence at later time. The number of IBD-specific biomarkers in *F. nucleatum*-experienced subjects is significantly higher than that in *F. nucleatum*-innocent subjects under non-IBD condition (*P* < 2.2e-16). The number of IBD-specific biomarkers was increased and that of non-IBD-specific biomarkers was decreased at the detection point of *F. nucleatum* and afterwards under non-IBD condition, leading to an alteration of microbiome. Similarly, the number of non-IBD-specific biomarkers in *F. nucleatum*-experienced subjects is significantly lower than that in *F. nucleatum*-innocent subjects (*P* = 3e-15). The number of IBD-specific biomarkers was not much changed before and after the detection of *F. nucleatum* under non-IBD condition (Fig. 4e). These results suggested that experience of *F. nucleatum* should be tightly linked with IBD development.

The association of biomarker species with *F. nucleatum* was also assessed by calculating Spearman’s correlation coefficients. All 14 non-IBD biomarkers were negatively correlated with *F. nucleatum,* having very significant enrichment *p*-values, and IBD biomarkers showed mostly positive correlation with some exceptions (Fig. 4f). Collectively, the absolute correlation coefficient of a certain microbe with *F. nucleatum* had strong relationship with its enrichment *p*-values in either IBD or non-IBD conditions (*ρ* = 0.33, *P* = 3.8e-15; Fig. 4f).

When the longitudinal abundance of the biomarker species was examined, two representative marker species of non-IBD condition, *Alistipes shahii* and *Alistipes putridinis*, showed the decreasing patterns of abundance along the X-axis standing for the proximal weeks to the detection point of *F. nucleatum.* In contrast, the abundance of IBD markers like *Clostridium symbiosum* and *Clostridium bolteae* had opposite pattern, low at prior and high at posterior to *F. nucleatum*-detection along the temporal axis (Fig. 4g). The abundance of these four biomarkers was significantly changed only in IBD condition, which means that the perturbation in key microbes’ abundance should be accompanied by chronic inflammation. Besides, two additional IBD markers, *Flavonifractor plautii* and a unclassified species in *Oscillibater* genus, and three non-IBD markers, *Alistipes finegoldii*, *Roseburia hominis*, *Roseburia inulinivorans,* exhibited similar patterns of abundance changes over time (Additional file 8: Figure S4).

### Microbial destabilization after *F. nucleatum* detection

Homeostasis of human gut microbiota is a sort of indicators of human health and understanding of their behavior is important for diagnosis and prevention of disease states. The microbial imbalance, called dysbiosis, is believed to cause or be associated with several metabolic and inflammatory diseases [37, 38]. To see whether *F. nucleatum* experience is associated with long-term stability of microbiome, we examined intra- and inter-individual alterations of microbiome in chronological order relative to the detection point of *F. nucleatum.*

**Fig. 5:**
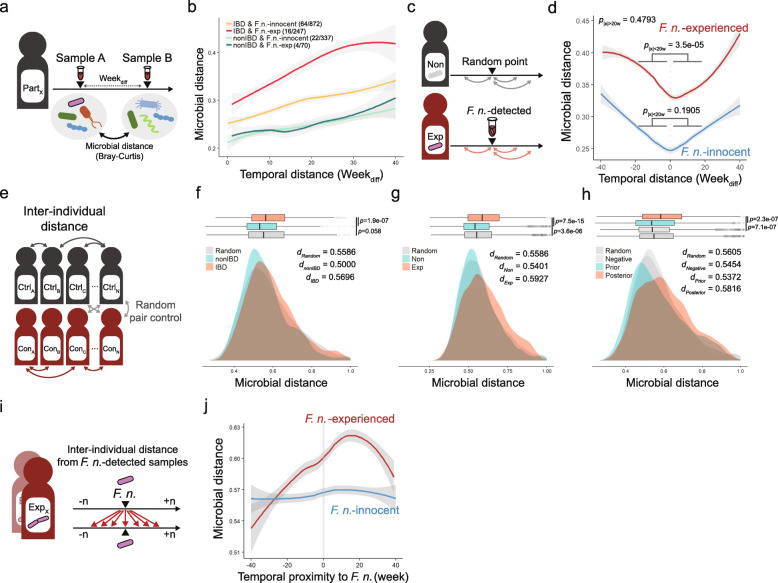
*F. nucleatum* experience is associated with microbial destabilization. **a** Analytic scheme for calculating intra-individual stability of microbiome. **b** Intra-individual dissimilarity of microbiome with different time intervals facetted by *F. nucleatum* experience and inflammatory conditions. Numbers of subjects and samples shown in parenthesis (# of subjects/ # of samples) **c** Analytic scheme for calculating intra-individual stability of microbiome with fixed initial point. **d** Intra-individual dissimilarity of microbiome with fixed initial time point. **e** Analytic scheme for calculating inter-individual dissimilarity of microbiome. **f-h** Inter-individual dissimilarity of microbiome by inflammatory conditions, *F. nucleatum* experience, and temporal distribution toward *F. nucleatum* observation, respectively. **i** Analytic scheme for calculating inter-individual dissimilarity of microbiome with fixed initial point. For *F. nucleatum*-innocent control, initial points were randomly selected. **j** Inter-individual dissimilarity of microbiome by temporal proximity to *F. nucleatum*

Intra-individual dissimilarity of microbiome was measured by pairwise Bray-Curtis distance after random sampling in a given participant (Fig. 5a). Consistent with the previous findings, IBD subjects regardless of *F. nucleatum-*experience, showed higher microbial dissimilarity than non-IBD subjects at any given time intervals, supporting that IBD is related with microbial destabilization [34]. By calculating the microbial distance, the microbiomes of IBD patients who have experienced *F. nucleatum* were verified to be more unstable than those of *F. nucleatum*-nonexperienced group (Fig. 5b). The temporal microbial stability was compared between before and after detection of *F. nucleatum* (Fig. 5c). *F. nucleatum*-experienced subjects showed significant dissimilarity between earlier time and later time points in *F. nucleatum*-experience samples (*P*_|x| < 20w_ = 3.5e-05), whereas *F. nucleatum*-innocent control did not (*P*_|x| < 20w_ = 0.1905) (Fig. 5d). Individual alterations in microbiome were traced over the time, resulting that four IBD subjects (C3009, H4015, M2034, and P6009) among 16 *F. nucleatum*-experienced IBD subjects showed dramatic microbial shift but four *F. nucleatum*-experienced non-IBD subjects did not (Additional file 9: Figure S5).

Two different participants under the same condition were randomly selected to estimate inter-individual microbial distance (Fig. 5e). The dissimilarity between IBD patients was higher than non-IBD subjects (*d*_IBD_ = 0.5696, *d*_non-IBD_ = 0.5000, *P* = 1.9e-07; Fig. 5f), and that of *F. nucleatum*-experienced subjects was also higher than non-experienced ones (*d*_exp_ = 0.5927, *d*_non-exp_ = 0.5401, *P* = 7.5e-15; Fig. 5g). The microbial distance on the temporal distribution was higher in samples posterior than prior to *F. nucleatum*-detection (*d*_posterior_ = 0.5816, *d*_*prior*_ = 0.5372 *P* = 2.3e-07; Fig. 5h). When one *F. nucleatum*-detected sample was compared with samples of different *F. nucleatum*-experienced subjects, the inter-individual microbial distance was gradually elevated until 20 weeks after *F. nucleatum* detection (Fig. 5i, j).

Collectively, these results suggested that highly variable microbiome might be pre-established in *F. nucleatum*-colonizing environment, and potentiate dysbiosis upon chronic inflammation. On the other hand, a convergent microbiome before *F. nucleatum* detection become unstable and divergent along with *F. nucleatum* occurrence, possibly leading to the formation of pathogenic microbiome.

### Identification of classifier microbes for *F. nucleatum* detection

**Fig. 6:**
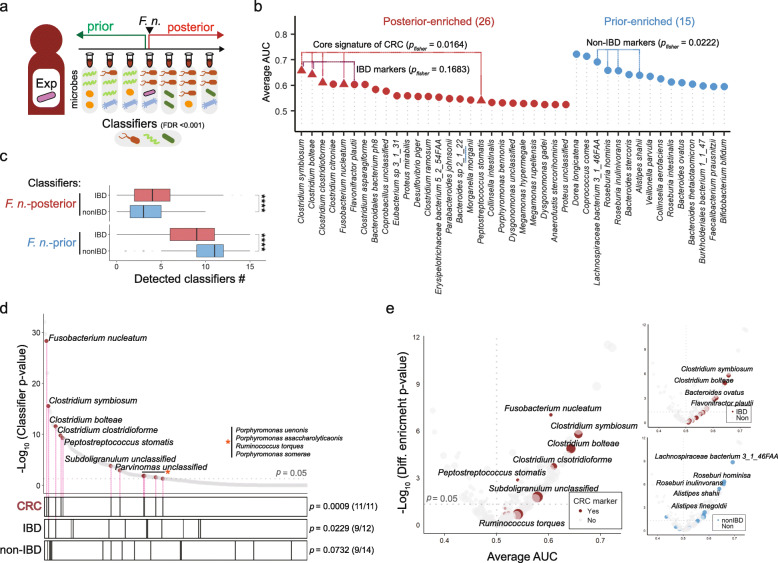
*F. nucleatum*-oriented dynamics is informative of capturing biomarkers for IBD or CRC. **a** Schematic illustration of screening classifier species in *F. nucleatum*-experienced subjects. **b** List of classifier microbes enriched in *F. nucleatum*-posterior or prior samples. Fisher’s exact test was performed. Triangles are CRC signature species. **c** Number of detected posterior- or prior-enriched classifiers in IBD or non-IBD samples. (Wilcoxon test. **** < 0.0001). **d** Classifying significance of microbes in *F. nucleatum*-experienced subjects. Red circle indicated CRC signature species detected in *F. nucleatum*-experienced subject at least 5 times. Fisher’s exact test was performed. Gray dotted line indicated *p*-value = 0.05. **e** Average AUC of microbes and their logarithmic *p*-value for differential enrichment in *F. nucleatum*-posterior (upper right) or -prior samples (lower right)

To identify representative microbes for *F. nucleatum* detection, all 317 samples from *F. nucleatum*-experienced subjects (16 IBD and 4 non-IBD participants) were partitioned and 258 microbes were initially screened following the procedure described in Methods. Among them, 41 significant species were predicted as “classifiers” for *F. nucleatum* by multiple logistic regression analysis (False discovery rate (FDR) < 0.001) (Fig. 6a). These classifier microbes were divided into two groups, 15 and 26 species enriched in samples prior and posterior to *F. nucleatum-*detection, respectively (Fig. 6b, Additional file 10: Table S5). The posterior-enriched classifiers, including 3 IBD marker species, were favorably found in IBD samples, and the prior-enriched classifier with 4 non-IBD marker species were preferentially observed in non-IBD samples (Fig. 6b, c).

A recent fecal metagenome analysis suggested 29 core signature bacteria enriched in CRC metagenomes including three *F. nucleatum* strains [39]*.* Among them, 18 CRC signature species were also observed in our dataset, and most of them (14 out of 17 signatures except *F. nucleatum*) were positively correlated with *F. nucleatum* (Additional file 11: Table S6). The five CRC signature species including three *Clostridium* species (*C. symbiosum*, *C. bolteae, C. clostridioforme*)*, F. nucleatum*, and *Peptostreptococcus stomatis* were overlapped with potent *F. nucleatum*-posterior classifiers (Area under the curve (AUC)_*C. sym.*_ = 0.6574, AUC_*C. bolt.*_ = 0.6427, AUC_*C. clostri.*_ = 0.6102, AUC_*F. nuc.*_ = 0.6043, AUC_*P. sto.*_ = 0.5406, *P*_CRC_ = 0.0164; Fig. 6b). Especially, *C. symbiosum* proposed as a potent fecal biomarker for CRC was the top *F. nucleatum*-posterior classifier in our study [40].

Considering discriminative property of microbial markers detected more than 5 times in *F. nucleatum*-experienced subjects, all 11 CRC biomarkers could successfully distinguish samples prior to *F. nucleatum*-detection from ones posterior to *F. nucleatum*-detection (*P*_CRC_ = 0.0009). Likewise, a majority of IBD and non-IBD markers (9 out of 11 and 9 out of 14, respectively) showed a discriminative power (*P*_IBD_ = 0.0229, *P*_non-IBD_ = 0.0732) (Fig. 6d). Most biomarkers identified in this study exhibited significant discriminative power for *F. nucleatum* detection and were differentially enriched in samples either prior or posterior to *F. nucleatum*-detection, supporting that *F. nucleatum*-oriented approach has an advantage to the effective identification of biomarkers (Fig. 6e).

### Estimation of *F. nucleatum* experience and dysbiosis level in *F. nucleatum*-innocent subjects

**Fig. 7:**
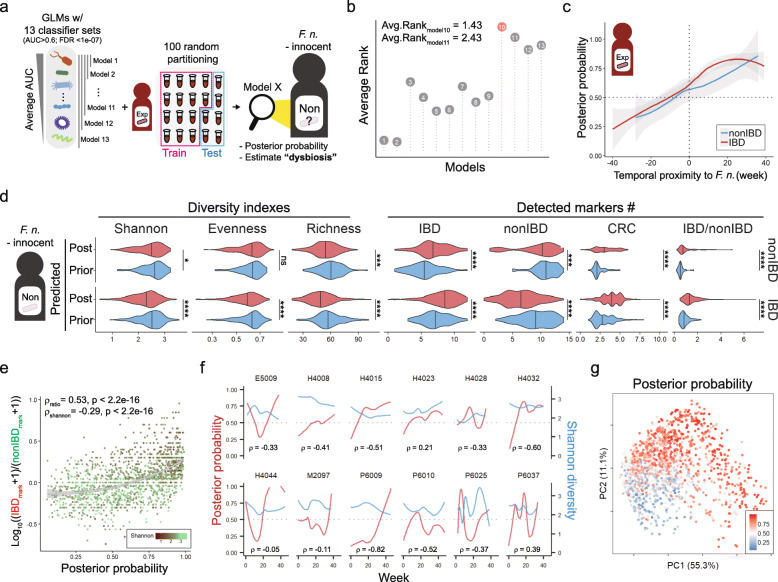
Estimation of *F. nucleatum*-experience and dysbiosis level in *F. nucleatum*-innocent subjects. **a** Schematic illustration of constructing multiple generalized linear regression model **b** Average rank of model performance. **c** Model validation using *F. nucleatum*-experienced subjects. Line color indicates inflammatory condition of subjects. **d** Characterization of predicted *F. nucleatum*-posterior or -prior groups in *F. nucleatum*-innocent subjects. Wilcoxon test. ns indicates non-significant (*p*-value> 0.5), ** *p* < 0.01, *** *p* < 0.001, **** *p* < 0.0001 **e** Correlation between posterior probability and IBD/non-IBD marker ratio or Shannon diversity. Dot color indicates Shannon diversity. Spearman correlation between two parameters and its significance was described at the top of scatter plot. **f** Intra-individual change of posterior probability of Shannon diversity in the top 12 dynamic individuals. Pearson correlation coefficients between posterior probability and Shannon diversity were shown. **g** Posterior probability of whole samples in PCoA plot

A prediction model was constructed to estimate the probability of experiencing *F. nucleatum* with top 13 potent classifiers satisfying average AUC > 0.6 and FDR < 1e-07 (Fig. 7a). The constructed generalized linear modeling (GLM) was tested with 100 randomly partitioned training datasets and the 10th GLM was chosen as the best model for examining the level of dysbiosis by considering average ranks in AUC, Akaike information criterion (AIC), accuracy, sensitivity, precision, and specificity (Fig. 7b, Additional file 12: Figure S6a-f). The 10 species used for building the 10th GLM were *Dorea longicatena, Coprococcus comes, Lachnospiraceae* bacterium 3_1_46FAA*, Clostridium symbiosum, Roseburia hominis, Roseburia inulinivorans, Alistipes shahii, Bacteroides stercoris, Clostridium bolteae*, and *Veillonella parvula* in descending order of mean AUC (Additional file 10: Table S5). When applying this model to *F. nucleatum*-experienced subjects for validation, the probability of experiencing *F. nucleatum*, so called “posterior probability”, was gradually increased and reached a decision threshold of 0.5 just before detection point of *F. nucleatum*, which means that this model can successfully predict the exact point of *F. nucleatum* detection (Fig. 7c). Strikingly, this model was still effective even when 86 *F. nucleatum*-innocent subjects were separated by predicted posterior probability and inflammation status. Samples with predicted posterior probability above 0.5 showed decreased alpha-diversity, increased number of biomarkers for IBD and CRC, and decreased number of non-IBD biomarkers indicating clear manifestations of dysbiosis (Fig. 7d, Additional file 12: Figure S6g). The posterior probability was correlated negatively with Shannon diversity and positively with the ratio of IBD to non-IBD markers (Spearman correlation, ρ_shannon_ = − 0.29, ρ_ratio_ = 0.53; Fig. 7e). There was a negative correlation between microbial diversity and the posterior probability when examined in the most 12 “dynamic” subjects with high variance in posterior probability (Fig. 7f, Additional file 13: Figure S7). Especially, several IBD patients including E5009, H4015, H4032, H4044, P6009, P6010, and P6025, displayed dramatic microbial shift as the posterior probability increased. Additionally, the negative correlation could be further generalized to more subjects in 70th percentile from the highest variance in posterior probability (Additional file 14: Figure S8). The samples with low posterior probability were located in the lower left side of the plot but the samples with high probability were scattered, indicating that our prediction model explained microbial variance properly (Fig. 7g).

The validity of our prediction model was further strengthened through its application with the independent metagenomic data from HMP phase I database generated by analyzing fecal samples of healthy population [41]. The best GLM model above was applied to 82 filtered samples out of 251 samples, where no samples contained *F. nucleatum* as expected. Consistently with the previous results, healthy microbiome showed a broad range of *F. nucleatum-*posterior probability and the posterior probability was negatively correlated with Shannon diversity (Additional file 15: Figure S9a, b). The samples predicted as *F. nucleatum*-posterior or -prior group were examined and *F. nucleatum*-posterior group was typically characterized by decreases in three indices of microbial alpha diversity (richness, evenness, and Shannon diversity), increase in the prevalence of IBD and CRC biomarkers, and significant decrease of non-IBD biomarker (Additional file 15: Figure S9c). These results strongly supported that the 10 classifier species screened by their longitudinal dynamics to *F. nucleatum* could predict gut dysbiosis even in healthy individuals.

### Application of potential biomarkers to the evaluation of fecal microbiome

**Fig. 8:**
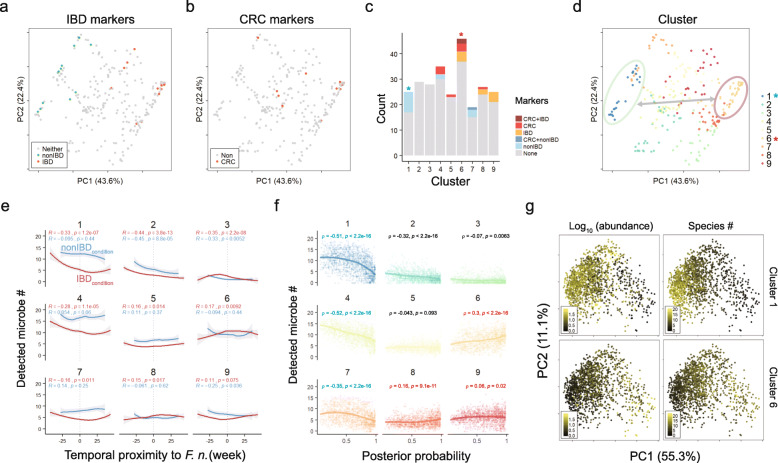
Clustering all detected microbes based on longitudinal distribution. **a** Distribution of IBD/non-IBD marker species on PCA plot. Euclidean distances between species were measured. **b** Distribution of CRC marker species. **c** K-mean clustering of microbes and biomarkers. Blue star marks for cluster 1 and red star for cluster 5 **d** Microbial distribution by clusters. Clusters 1 and 6 were encircled. **e** Detected number of cluster component per sample along temporal proximity to *F. nucleatum* observation. Line color indicates sample condition. Spearman correlation and its significance were calculated. **f** Correlation between posterior probability and the number of detected microbes by clusters. Spearman correlation and its significance were calculated. **g** Distribution of clusters 1 and 6 in PCoA plot of samples. Logarithmic abundance and the number of detected species were displayed

To classify the microbial distribution, we considered 5 following criteria; 1) Spearman co-abundance correlation with *F. nucleatum*, 2) enrichment in IBD condition, 3) enrichment in *F. nucleatum*-experienced subjects, 4) enrichment in samples posterior to *F. nucleatum* detection, 5) discriminative significance for *F. nucleatum* detection. The biomarker species for IBD and non-IBD conditions were distinguishable in principal component analysis (PCA) plot, and the CRC signature species were closely related with IBD biomarkers (Fig. 8a, b).

The effectiveness of our IBD/non-IBD biomarkers as well as CRC markers in the longitudinal analysis was validated by K-means clustering of all microbes. Among 9 clusters, cluster 1 harbored most non-IBD biomarkers (8/14) and cluster 6 had five CRC and six IBD biomarkers, where *C. symbiosum* and *C. bolteae* belong to both sides. Moreover, cluster 6 held many known opportunists such as *Clostridium difficile, Enterococcus faecalis, Enterococcus faecium*, *Escherichia coli, Haemophilus haemolyticus*, *Saccharomyces cerevisiae*, and *F. nucleatum* (Additional file 16: Table S7). Cluster 4 had both CRC and non-IBD markers. Cluster 8 and 9 contained IBD markers (Fig. 8c). Notably, the cluster 1 and 6 were separated far apart in PCA plot, and the cluster 8 and 9 were localized near cluster 6, which indicated that the biomarker species with a similar character formed intimate clusters (Fig. 8d).

The number of detected microbes along temporal proximity to *F. nucleatum* was decreased in clusters 1, 4, and 7 where non-IBD biomarkers were involved (Fig. 8e). The number of detected microbes increased in IBD condition of clusters 6 and 8, which had both CRC and IBD biomarkers. Interestingly, although the cluster 2 and 3 showed significant decrease in detected microbe number regardless of inflammatory conditions, they did not contain any biomarkers. In accordance with Fig. 4e, the number of dysbiosis-associated biomarkers changed in IBD condition. Clusters 1, 4, and 7 were negatively correlated with the posterior probability, but the clusters 6 and 8 were positively related (Fig. 8f). Furthermore, the clusters 1 and 6 exhibited a complementary distribution each other in terms of microbial abundance and detection frequency, which was confirmed in independent healthy dataset (Fig. 8g, Additional file 15: Figure S9d).

Taken together, our work illuminated previously unrecognized knowledge on the early gut dysbiosis in the context of chronological dynamics of microbiome by focusing on the opportunistic colonization of *F. nucleatum*. It is noteworthy that even a rare microbial species under a certain condition could be used as an indicator for predicting a perturbation in the future event, as shown with *F. nucleatum*-focused longitudinal modeling. Although further experiments were needed to verify physiology of the classifier microbes, we expected that analysis on chronological alteration of microbiome would be greatly helpful for biomarker screening and diagnosis of microbiota-associated diseases.

## Discussion

Commensal microbiota in the healthy gut controls pathogens and pathobionts by direct interactions, stimulating host immunity, preventing their colonization [42]. Changes in microbial abundance reflect healthy and disease states. Previously, metagenomic biomarker discovery was performed by way of class comparison between two or more microbial communities [43]. However, enrichment or localization of microbiota in the intestine could be explained more clearly by tracking a group of prevalent and abundant species for the microbiota-associated chronic gut disorders rather than a single or a couple of rare opportunistic pathogens.

This study is the first trial to screen non-invasive biomarkers at species level, responsible for the early gut dysbiosis in a longitudinal view. Gut microbiota homeostasis is maintained under normal condition but unfavorable conditions may influence the microbial diversity, leading to gut dysbiosis [44]. Metagenomic profiling of IBD samples showed lower diversity than non-IBD samples, as expected. *F. nucleatum* is rarely found in gut microbiome and has been recently considered as a potential oncobacterium associated with human cancers. The longitudinal tracking of *F. nucleatum*-experienced subjects indicated that *F. nucleatum* might appear under gut microbiome perturbation toward a low microbial diversity. *F. nucleatum* was truly associated with biomarker species for IBD. Indeed, *C. symbiosum,* the top-ranked biomarker for *F. nucleatum*-marked dysbiosis in our study, was proposed as a potent fecal biomarker for CRC even superior to *F. nucleatum* [40]. Furthermore, among 15 prior-enriched classifier species, *Dorea longicatena* with the highest discrimination ability (AUC = 0.7224) was recently proposed as one of potential probiotics for metabolic disorder and also reported to be over-represented in remissive CD patients after ileocolonic resection when compared to recurrent cases [45, 46]. *Coprococcus comes* (AUC = 0.7143) was reported to show a down-regulation in CRC patients, and three *Roseburia* species including *R. hominis*, *R. inulinivorans*, and *R. intestinalis* (AUC_*R.hom.*_ = 0.6594, AUC_*R.inul.*_ = 0.6576, AUC_*R.intest.*_ = 0.6140)*,* were well-documented to shape beneficial gut microflora by fermenting dietary polysaccharides [47–50]. Even if Lloyd-Price et al. reported a group of microbes such as *Prevotella copri* as a representative species for microbial shift in non-IBD condition, the shift itself was not enhanced with chronic inflammation and the biomarkers for the shift did not tell whether they represent favorable alterations or not [34]. In our analysis, *P. copri* appeared in *F. nucleatum*-experienced non-IBD subjects after *F. nucleatum* detection with marginal significance, implying its pro-dysbiotic property. Certain microbes such as *Bacteroides uniformis, Bacteroides ovatus*, and most *Veillonella* species, characterized by their positive association with gastrointestinal diseases, pre-colonize before *F. nucleatum* appearance (Additional file 16: Table S7) [47, 51–55]. In addition, many *Streptococcus* and *Bifidobacterium* genus were differentially enriched before *F. nucleatum*, indicating that the particular community of microbes might be required for *F. nucleatum* colonization.

Our *F. nucleatum*-based model effectively identified changes in gut microbiome when tested with an independent dataset from healthy individuals, which suggests that chronological dynamics of microbiome may be conserved in human population. Further analysis should be conducted to identify microbial pathways that favor pro-dysbiotic gut, which would enable to understand biology of gut homeostasis.

## Conclusions

This study revealed that opportunistic appearance of *F. nucleatum* in fecal metagenome reflected early establishment of dysbiotic environment in the gut. Distribution of IBD and non-IBD biomarkers was significantly altered by *F. nucleatum* experience. Samples collected after *F. nucleatum* appearance showed high intra- and inter-individual dissimilarity, indicating that occurrence of *F. nucleatum* might serve as a trigger for perturbation and increased divergence of microbiome. The 41 classifier species, predicted discriminators for *F. nucleatum* occurrence, were identified and their effectiveness was validated in *F. nucleatum*-innocent subjects. They included known core signature species for CRC and marker microbes for health gut as well. The classifier-based prediction model successfully estimated microbial dysbiotic state and colonization of diseases-associated microbes. The potential probability of experiencing *F. nucleatum* was significantly associated with the distribution of biomarkers, microbial diversity and inter-personal divergence. To suggest potential biomarkers for symbiosis and dysbiosis, microbes were classified by their distribution characteristics. Our results highlight a novel layer of information on microbial dynamics during early gut dysbiosis and can be used to develop conditional biomarkers focused on a specific microbe.

## Methods

### Data curation and taxonomy assignment

A total of 1638 fecal metagenomic samples (1338 HMP data and 300 HMP pilot data), longitudinally collected from 130 participants were downloaded from IBDMD (https://ibdmdb.org/) [33]. To certify longitudinal sampling, the data from 106 participants (80 IBD patients and 26 non-IBD participants) who provided fecal samples more than 5 times was considered. Technical replicates were not used in this study. After filtering, metagenomic analysis of 1560 fecal sample data (243 HMP pilot and 1317 HMP) from 106 participants was performed at species-level resolution by MetaPhlAn2 [35]. To improve taxonomic resolution of metagenomic data and to reduce outlier-driven statistical distortion, the following three conditions for quality control were applied: 1) Species level explains more than 90% of total microbiome. 2) Total bacterial abundance accounts for 70% of whole metagenome. 3) Minimum number of bacterial species is greater than 17. A total of 1526 samples were selected for the further analysis.

For model validation, cross-sectional metagenomic data was obtained from HMP data portal (https://portal.hmpdacc.org/). Among 251 fecal samples that were collected from HMP phase I, one third of the samples (84 samples) were randomly selected and processed with MetaPhlan2. After excluding two samples that failed to satisfy quality criteria, the probability of *F. nucleatum* experience for the remaining 82 samples were measured using our prediction model. Simple manifest file, metadata, and microbial abundance matrix for the validation dataset were included in Additional file 17: Table S8.

### Sample classification based on the diseases severity

Simple complex colitis activity index (SCCAI) and Harvey-Bradshaw index (HBI) were available in 413 UC-derived samples and 650 CD-derived samples, respectively (Additional file 17: Table S8). HBI is a simpler version of the Crohn’s disease activity index (CDAI), which enables patients to self-diagnose the diseases severity. We classified samples based on the disease severity, considering the following guidelines: 1) Remission: SCCAI ≤2, and HBI ≤3; 2) Border: 3 ≤ SCCAI ≤5, and 4 ≤ HBI ≤7; 3) Active: SCCAI ≥6, and HBI ≥8 [56, 57].

### Principal coordinate analysis

Microbial abundance data was log10-transformed after adding 1e-05 pseudo-abundance, Then, integer 5 was added to remove negative values and Bray-Curtis dissimilarity was calculated between samples. Principal coordinates analysis was conducted using vegdist function in vegan R package and cmdscale function in stat R package. To examine whether samples are distinguished by their metadata, we performed analysis of variance (ANOVA) for comparing IBD vs. non-IBD, UC vs. CD, and *F. nucleatum*-innocence vs. -experience. For visualization of distributional variance of microbes, PCA using Euclidean distance was performed using five features as described below: 1) *P*-value for Spearman abundance correlation coefficients with *F. nucleatum,* 2) *P*-value for the differential enrichment in IBD condition, 3) *P*-value for the differential enrichment in samples from *F. nucleatum*-experienced subjects, 4) *P*-value for the differential enrichment in samples after *F. nucleatum* detection, and 5) *P*-value for discriminating samples posterior to *F. nucleatum* detection from those prior to *F. nucleatum* detection in 100 random partitioned datasets. Because the significances for *F. nucleatum*-posterior enrichment and classifying samples were measured only for microbes detected in *F. nucleatum*-experienced subjects at least 5 times, 258 microbes out of 533 total species were analyzed and visualized in PCA plot.

### K-means clustering

To test if three conditions of samples (non-IBD, UC, and CD), were distinguishable by their microbial composition, we performed K-mean clustering using kmeans function in stat R package Microbial abundance matrix was added by 1e-05 pseudo-abundance and log10- transformed. Then, all samples were grouped into 3 clusters and tested whether each cluster was over-represented in particular conditions. The ORs of each condition to three clusters were calculated, and the highest values per condition were described: OR_nonIBD-C3_ = 4.42, OR_UC-C3_ = 2.30, OR_CD-C2_ = 2.15. With fixed random condition using set.seed (12345), 102 samples among 407 non-IBD samples, fell into the cluster 1, 56 samples into cluster 2, and 249 samples into cluster 3. CD samples were grouped by 309, 194, and 199 in each cluster, and UC samples were divided by 254, 68, and 95. These numbers were statistically compared by Fisher’s exact test and ORs. To classify the microbes based on their distributional features, we clustered 258 species that were detected at least 5 times in *F. nucleatum*-experienced subjects using K-means clustering. The best number of cluster was determined by vote using NbClust function in R package. Features on microbial dynamics were the same as previously described in PCoA method section above.

### Classification of samples based on F. nucleatum experience

Once *F. nucleatum* was detected in one subject for sample collection period, he/she was regarded as an experienced individual. Among 106 participants, 20 subjects (16 IBD patients and 4 non-IBD participants) have experienced *F. nucleatum* for a year. Even though one *F. nucleatum*-positive sample (sample ID: MSM9VZLZ; participant ID: M2083) was excluded in the sample curation step due to low species number, this subject was classified as *F. nucleatum*-experienced and included in the later analyses. Among 1526 samples, 317 samples (70 non-IBD and 247 IBD) were collected from *F. nucleatum*-experienced subjects, and 1209 samples (337 non-IBD and 872 IBD) were from *F. nucleatum*-nonexperienced (or –innocent) subjects. *F. nucleatum*-experienced samples were also categorized by temporal proximity toward *F. nucleatum*. If samples were collected within 4-weeks from *F. nucleatum*-detected points, they were classified as proximal ones and if not, distal ones.

### Screening microbial biomarker species for IBD and non-IBD condition

To identify microbial biomarkers that were differentially enriched in IBD or non-IBD conditions, we used a web-based linear discriminant analysis effect size (LEfSe) algorithm (http://huttenhower.sph.harvard.edu/galaxy/), which estimates not only the differential abundance of features among the classes but also the biological consistency within a same class [43]. Here, by setting IBD subtypes (UC and CD) as a sub-class of IBD, we could obtain common inflammatory biomarkers that changed similarly in both UC and CD conditions rather than showed specific alteration in UC or CD, which allows us to capture shared intestinal perturbation in two different inflammatory diseases. Significance thresholds of 0.05 were applied to both between-classes Krustal-Wallis test and pairwise within-classes Wilcoxon test. LDA score threshold was 2.5. Detailed results were included in an Additional file 7: Table S4.

### Microbial dissimilarity analysis

Pairwise microbial distance was calculated by Bray-Curtis dissimilarity equation. To calculate microbial dissimilarity within-an-individual, one subject was randomly selected for 10,000 times, from whom two samples were chosen. Then, temporal distance and microbial distance between the two samples were measured. According to metadata of the chosen subject, microbial distance was visualized by condition along temporal distance. For inter-individual dissimilarity test, the subjects were divided into three groups based on their classification categories such as inflammatory condition, *F. nucleatum* experience, or longitudinal distribution toward *F. nucleatum* observation, and each sample was picked up from two random subjects. As a control of inter-individual distance, two samples were randomly selected regardless of categories. To examine microbial composition by temporal proximity toward *F. nucleatum*-detected point, the *F. nucleatum*-detected samples was set as the initial point and the other random sample was selected from the same subject. Two randomly picked samples from a *F. nucleatum*-innocent subject were served as control.

### Screening classifier and construction of generalized linear models for dysbiosis prediction

To construct a prediction model for *F. nucleatum* experience, we first screened “classifier” microbes that distinguish *F. nucleatum*-prior from *F. nucleatum*-posterior samples. After partitioning 317 samples from *F. nucleatum*-experienced subjects 1000 times using a createDataPartition function in caret R package, a total of 258 microbes, identified at least 5 times across *F. nucleatum*-experienced subjects, were tested for their discriminative ability for samples prior or posterior to *F. nucleatum*-detection. The values of area under the Receiver Operating Character (ROC) curve (AUC) was calculated using roc function in pROC R package, and 41 significant species with average AUC value above 0.5 in multiple logistic regression models (FDR < 0.001) were regarded as classifiers. Here, to improve the number of samples, 41 *F. nucleatum*-detected samples were considered as *F. nucleatum*-posterior group. Detailed information for classifier species were included in an Additional file 10: Table S5.

Among 41 classifiers, top-13 potent classifiers except *F. nucleatum* (average AUC > 0.6 & classifying FDR < 1e-07) and inflammatory condition of subjects were used to construct a prediction model for the estimation of the probability of experiencing *F. nucleatum*. To find out the best set of classifiers, we added classifiers one by one from the top to the 13th in a decreasing order of average AUC, resulting in 13 different feature sets. In a similar way of classifier screening, samples from *F. nucleatum*-experienced subjects were divided into training and test set for 100 times using createDataPartition function, and multiple GLMs were generated (total 1300 models; one model/training set with 100 training sets and 13 feature combinations). The best performer was selected by averaging performance ranks of cross-validation AUC with training set, AUC with test set, Akaike information criterion (AIC), and four prediction statistics with decision threshold at 0.5 (accuracy, sensitivity, specificity, precision). The selected model number 10 was used for subsequent analysis.

## Supplementary information

**Additional file 1.** Table S1. Filtering step: removing replicated samples or participants with insufficient number of collections.

**Additional file 2.** Table S2. Quality control: removing samples with poor taxonomic assignment.

**Additional file 3.** Table S3. Basic information of participants.

**Additional file 4. **Figure S1. Microbial variation by sample categories. **(a)** Sex. **(b)** Disease severity. The severity was classified based on their diseases scores. **(c)** Participant. **(d)** Institutes. Five different institutes have collected fecal samples of IBD and non-IBD participants.

**Additional file 5. **Figure S2. Microbial diversity and human read fraction. (a) Pielou’s evenness, (b) Richness, (c) simple clinical colitis activity index (SCCAI) for UC, (d) Harvey-Bradshaw index (HBI) for CD, (e) Pielou’s evenness for *F. nucleatum*-experience, (f) Richness for *F. nucleatum*-experience.

**Additional file 6.** Figure S3. Low detection probability of opportunistic microbes. (a) Microbial abundance and its detection frequency in 44 duplicated samples, (b) Proportion of half-recovered species among total detected species, (c) Correlation between microbial abundance and detection number. Dot color indicates recovery rate of a certain microbe in pairs.

**Additional file 7.** Table S4. LEfSe biomarker screening results.

**Additional file 8. **Figure S4. Abundance changes for microbial biomarkers. (a) non-IBD markers, (b) IBD markers. Line color indicates sample conditions (red line for IBD, blue line for non-IBD). * indicates *p*-value < 0.05, ** *p* < 0.011, *** *p* < 0.001, **** *p* < 0.0001

**Additional file 9. **Figure S5. PCoA plot of 20 *F. nucleatum*-experienced subjects. Line color indicates temporal proximity to *F. nucleatum*.

**Additional file 10. **Table S5. Classifier species enriched prior or posterior to the detection point of *F. nucleatum*.

**Additional file 11. **Table S6. Correlation coefficients with *F. nucleatum* and multiple enrichment tests for global biomarker species of colorectal cancer (CRC).

**Additional file 12. **Figure S6. Model performance comparison and application into *F. nucleatum*-innocent samples. (a) AUC, (b) AIC, (c) accuracy, (d) sensitivity, (e) precision, (f) specificity, (g) The best model number 10 was applied to sample from *F. nucleatum*-innocent subjects. X-axis indicates participant ID. Blue indicates non-IBD and red indicates IBD.

**Additional file 13.** Figure S7. Individual alteration of microbiome in 12 dynamic subjects by inflammatory conditions and posterior probability. Line color indicates posterior probability.

**Additional file 14.** Figure S8. Intra-individual change of posterior probability and Shannon diversity in 70th percentile dynamic subjects. Pearson correlation coefficients were shown at the bottom of each participant panel.

**Additional file 15. **Figure S9. Model validation on independent healthy individuals. (a) Posterior probability of 82 fecal samples from healthy individuals. (b) Spearman correlation between posterior probability of *F. nucleatum* and Shannon diversity. (c) Microbial manifestations in putative *F. nucleatum*-prior or posterior samples. Wilcoxon rank sum test was performed. (d) prevalence of cluster 1 and 6 in validation dataset. * indicates *p*-value < 0.05, ** *p* < 0.011, *** *p* < 0.001.

**Additional file 16. **Table S7 Summary of microbial correlation with *F. nucleatum* and enrichment tests.

**Additional file 17.** Table S8. Metadata.

## Data Availability

The metagenome data was downloaded from IBDMD (https://ibdmdb.org/) and HMP data portal (https://portal.hmpdacc.org/). Detailed codes and input data used in this study can be found at GitHub (https://github.com/JW-Huh/F.nucleatum-project). Supplementary information is provided separately.

## Abbreviations

AUC: Area under the curve
CD: Crohn’s disease
CRC: Colorectal cancer
FDR: False discovery rate
*F. nucleatum*: *Fusobacterium nucleatum*
GLM: Generalized linear model
HBI: Harvey-Bradshaw index
HMP: Human Microbiome Project
IBD: Inflammatory bowel diseases
IBDMD: Inflammatory Bowel Disease Multi'omics Database
iHMP: Integrative Human Microbiome Project
LDA: Linear discriminant analysis
LEfSe: Linear discriminant analysis effect size
OR: Odd ratio
PCA: Principal component analysis
PCoA: Principal coordinate analysis
ROC: Receiver operating character
SCCAI: Simple clinical colitis activity index
UC: Ulcerative colitis
