# Supplementary material for: Opportunistic detection of Fusobacterium nucleatum as a marker for the early gut microbial dysbiosis
Source: BMC Microbiol. 2020 Jul 13;20:208. doi: 10.1186/s12866-020-01887-4 (PMC7359021; doi:10.1186/s12866-020-01887-4)
Supplement: Supplementary file 3 — Additional file 3. Table S3. Basic information of participants. [file 12866_2020_1887_MOESM3_ESM.docx]

|  | **Cohort #** | **Sex**  (Female; N; (%)) | **Consent age** | **Sample #** | **Collection days**  (Mean ± s.d.) |
| --- | --- | --- | --- | --- | --- |
| Non-IBD | 26 | 11 (42.3%) | 29.7 | 407 | 307.5 ± 38.4 |
| IBD | 80 | 41 (51.3%) | 26.9 | 1119 | 292.5 ± 39.5 |
| Total | 106 | 52 (49.1%) | 27.6 | 1526 | 296.2 ± 39.6 |

Table 1. Basic information of participants
