# Supplementary figures and images for: Opportunistic detection of Fusobacterium nucleatum as a marker for the early gut microbial dysbiosis
Source: BMC Microbiol. 2020 Jul 13;20:208. doi: 10.1186/s12866-020-01887-4 (PMC7359021; doi:10.1186/s12866-020-01887-4)

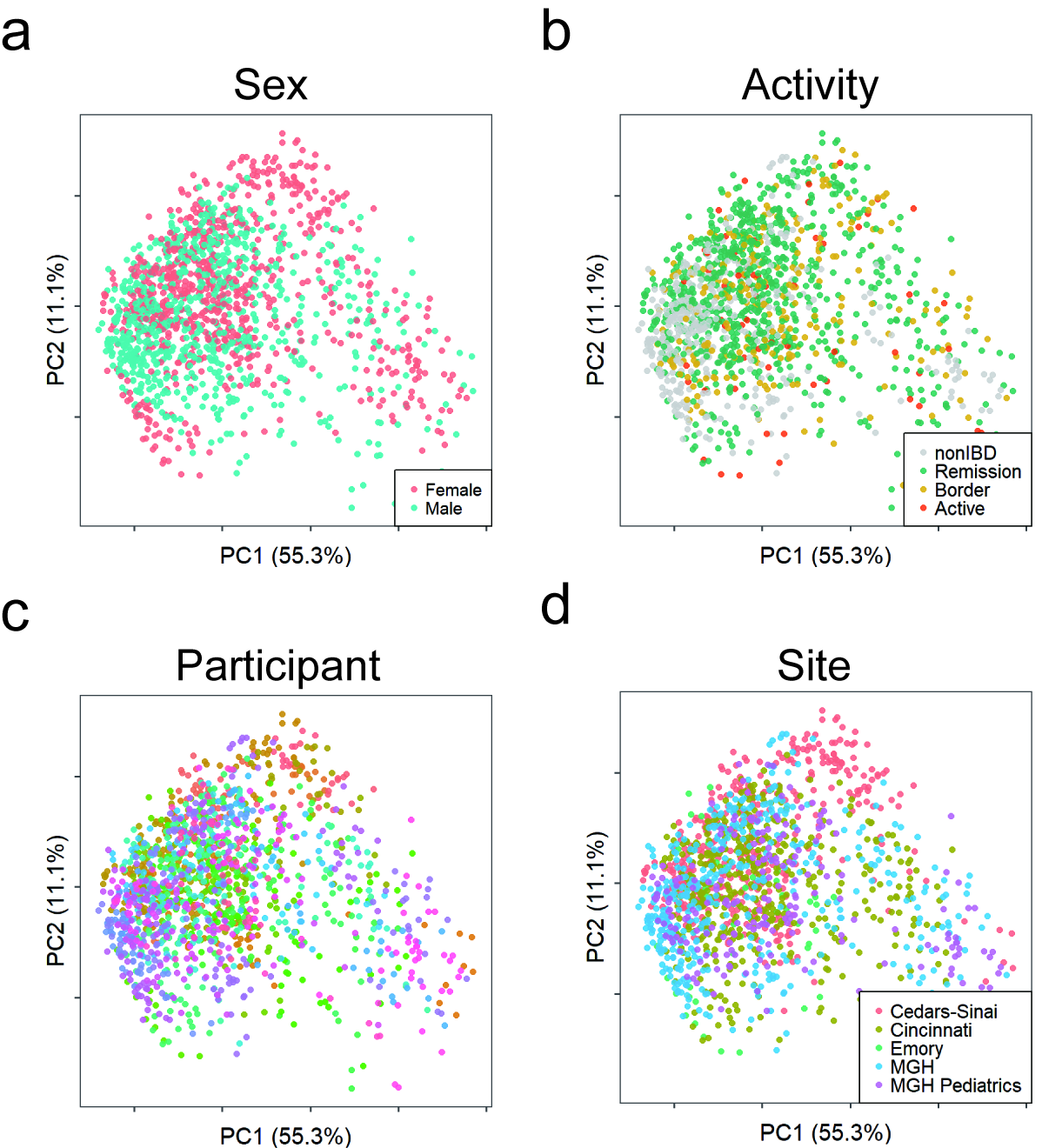

Supplement: Supplementary file 4 — Additional file 4. Figure S1. Microbial variation by sample categories. (a) Sex. (b) Disease severity. The severity was classified based on their diseases scores. (c) Participant. (d) Institutes. Five different institutes have collected fecal samples of IBD and non-IBD participants. [file 12866_2020_1887_MOESM4_ESM.tif]

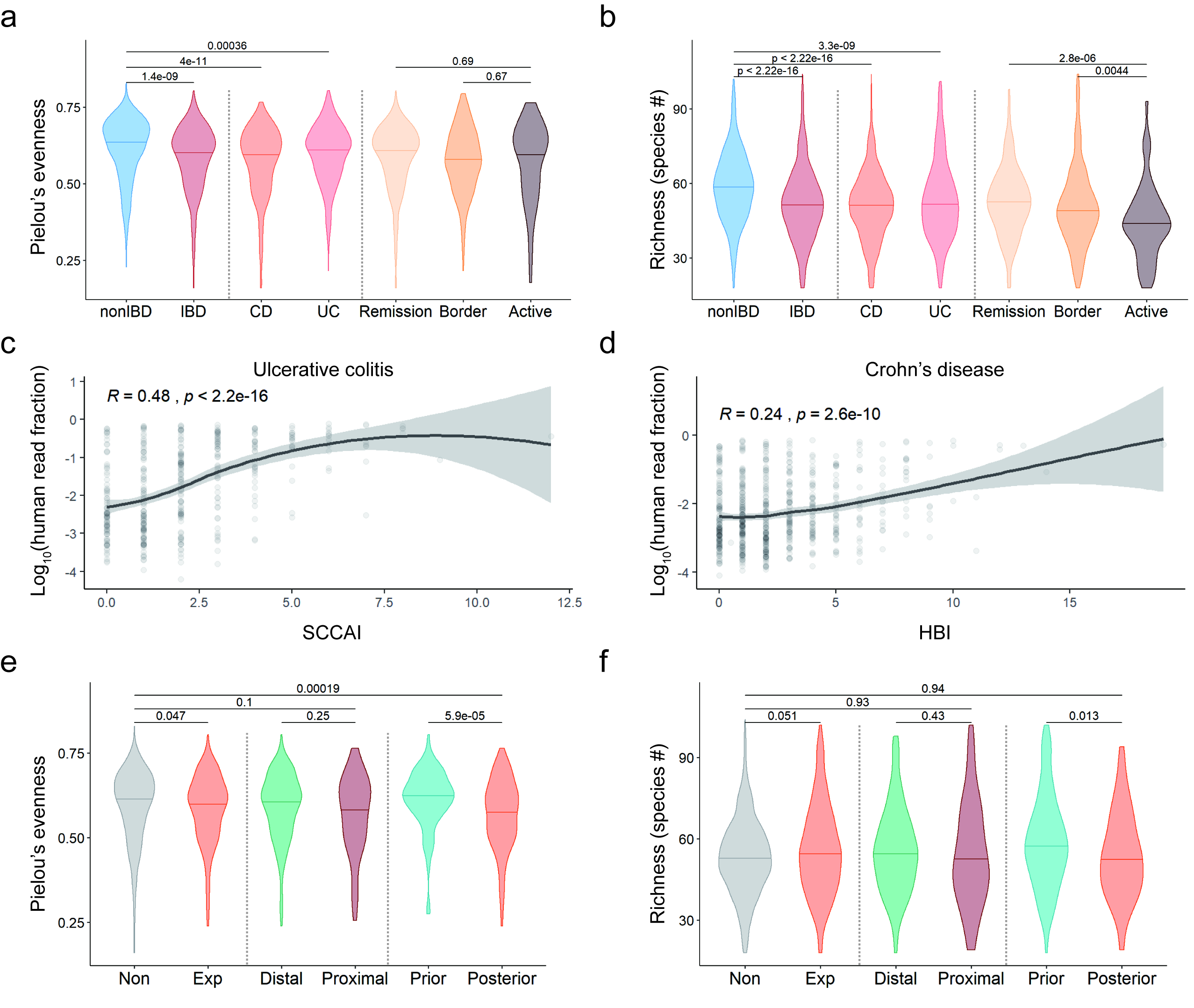

Supplement: Supplementary file 5 — Additional file 5. Figure S2. Microbial diversity and human read fraction. (a) Pielou’s evenness, (b) Richness, (c) simple clinical colitis activity index (SCCAI) for UC, (d) Harvey-Bradshaw index (HBI) for CD, (e) Pielou’s evenness for F. nucleatum-experience, (f) Richness for F. nucleatum-experience. [file 12866_2020_1887_MOESM5_ESM.tif]

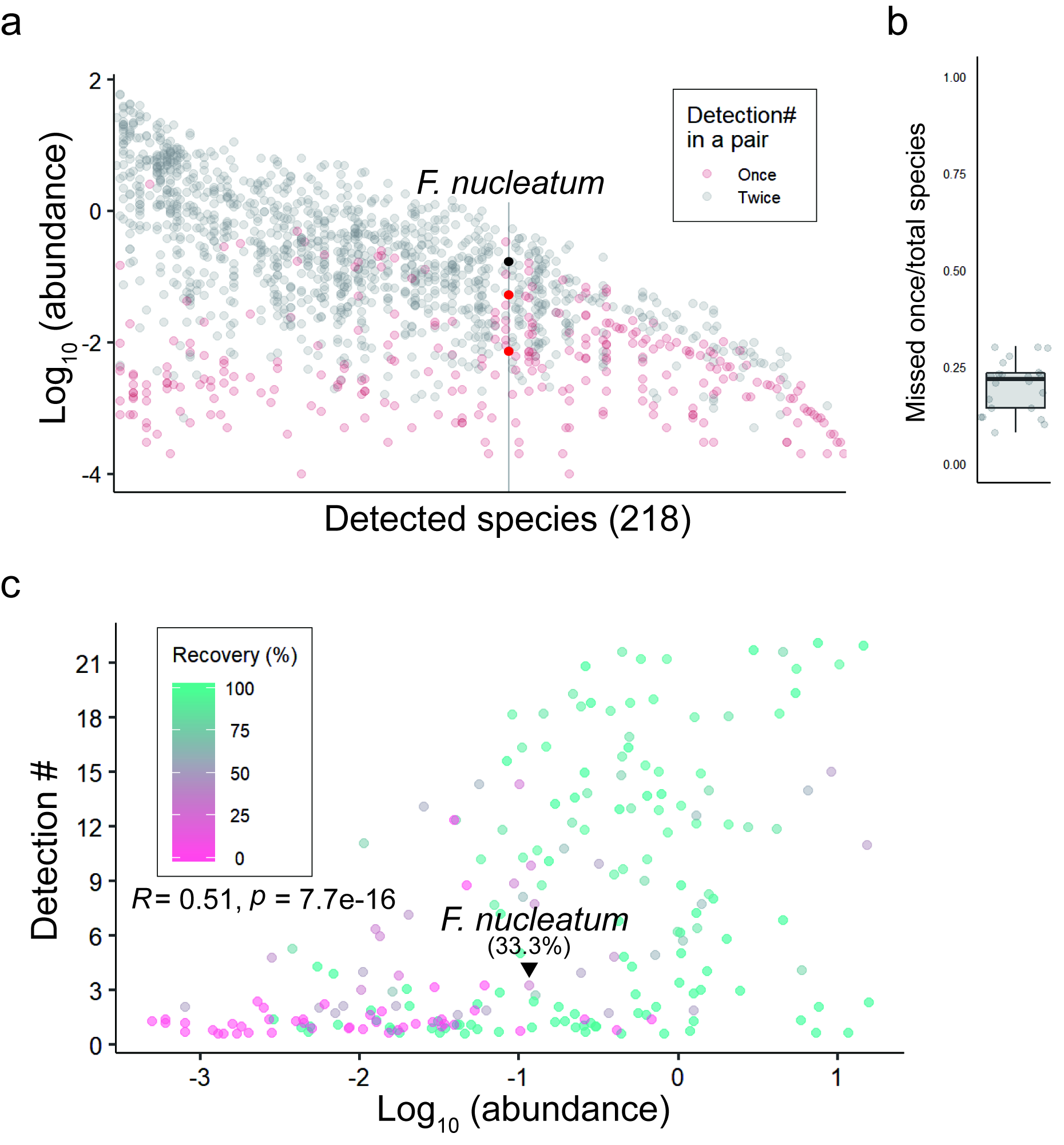

Supplement: Supplementary file 6 — Additional file 6. Figure S3. Low detection probability of opportunistic microbes. (a) Microbial abundance and its detection frequency in 44 duplicated samples, (b) Proportion of half-recovered species among total detected species, (c) Correlation between microbial abundance and detection number. Dot color indicates recovery rate of a certain microbe in pairs. [file 12866_2020_1887_MOESM6_ESM.tif]

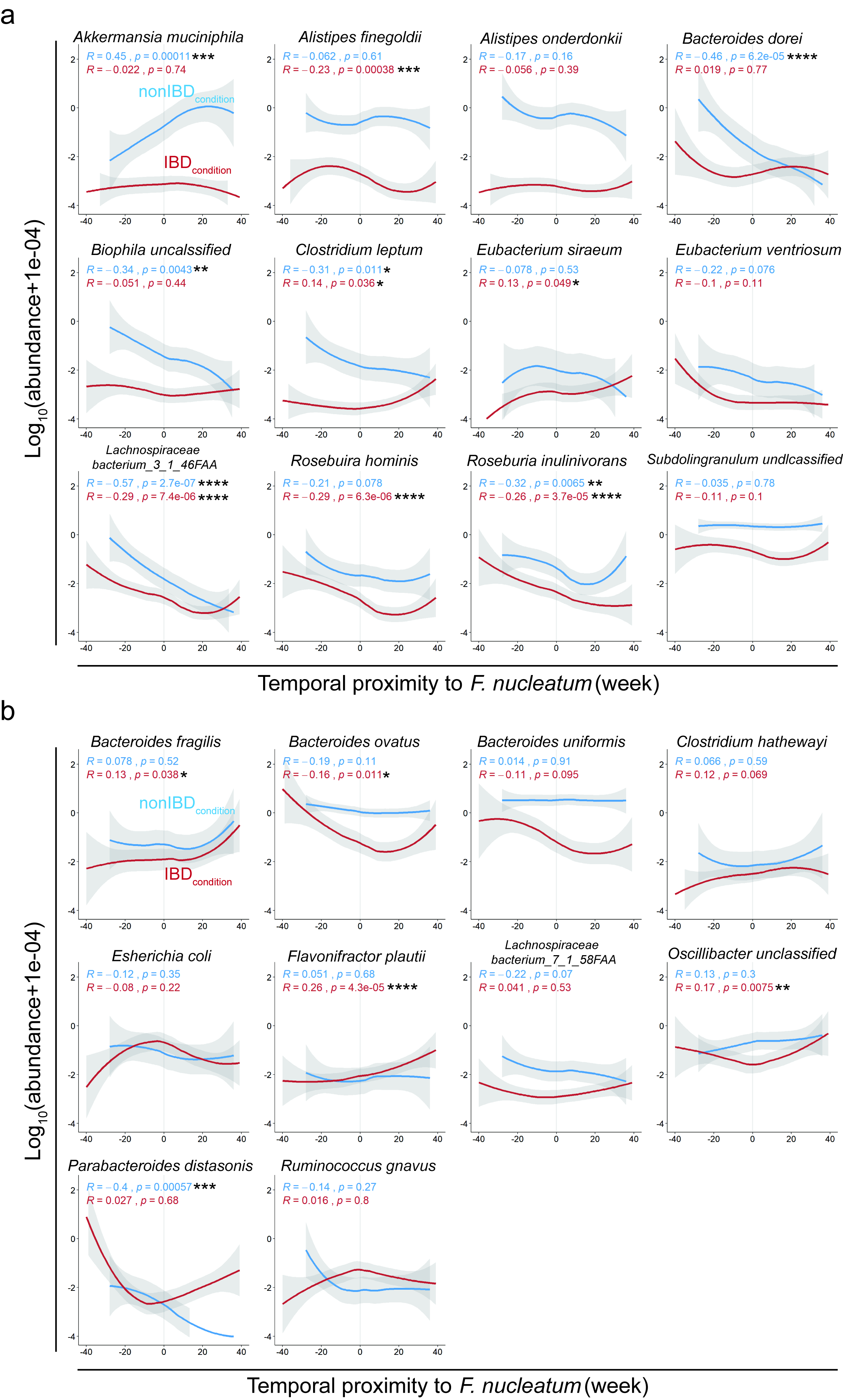

Supplement: Supplementary file 8 — Additional file 8. Figure S4. Abundance changes for microbial biomarkers. (a) non-IBD markers, (b) IBD markers. Line color indicates sample conditions (red line for IBD, blue line for non-IBD). * indicates p-value < 0.05, ** p < 0.011, *** p < 0.001, **** p < 0.0001 [file 12866_2020_1887_MOESM8_ESM.tif]

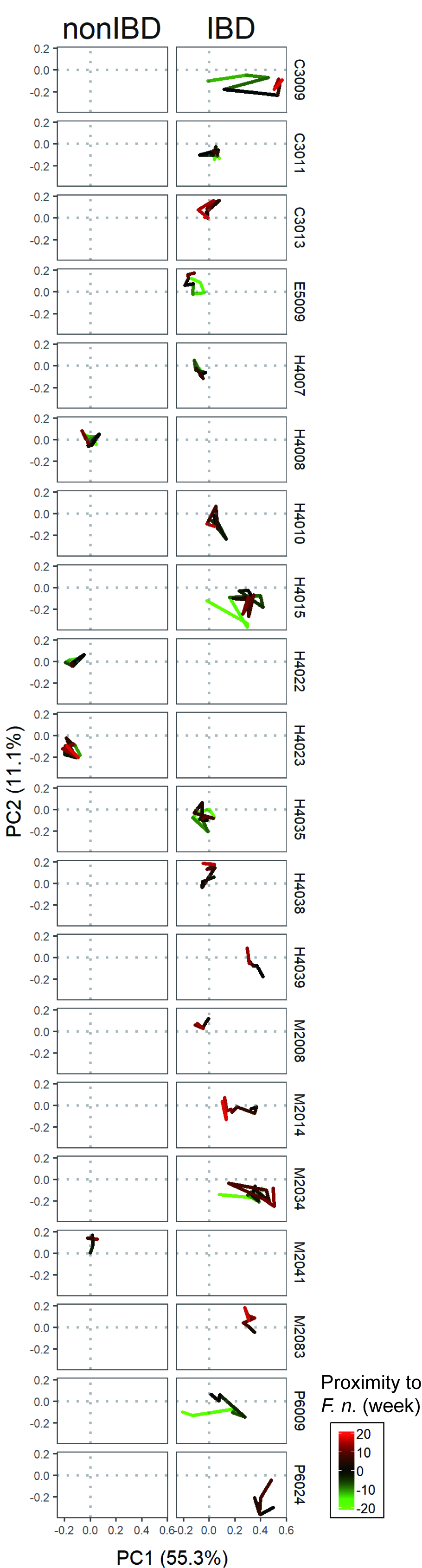

Supplement: Supplementary file 9 — Additional file 9. Figure S5. PCoA plot of 20 F. nucleatum-experienced subjects. Line color indicates temporal proximity to F. nucleatum. [file 12866_2020_1887_MOESM9_ESM.tif]

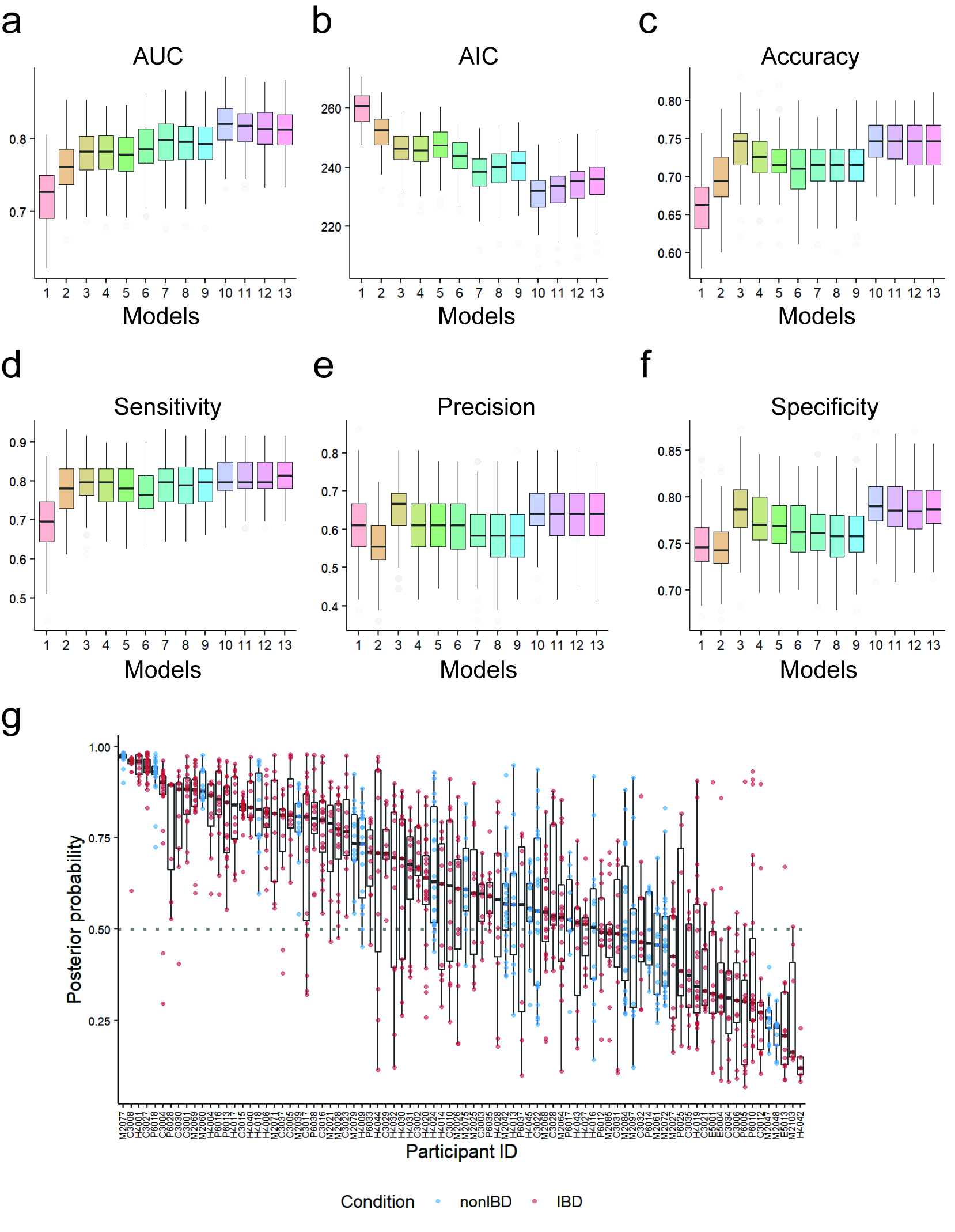

Supplement: Supplementary file 12 — Additional file 12. Figure S6. Model performance comparison and application into F. nucleatum-innocent samples. (a) AUC, (b) AIC, (c) accuracy, (d) sensitivity, (e) precision, (f) specificity, (g) The best model number 10 was applied to sample from F. nucleatum-innocent subjects. X-axis indicates participant ID. Blue indicates non-IBD and red indicates IBD. [file 12866_2020_1887_MOESM12_ESM.tif]

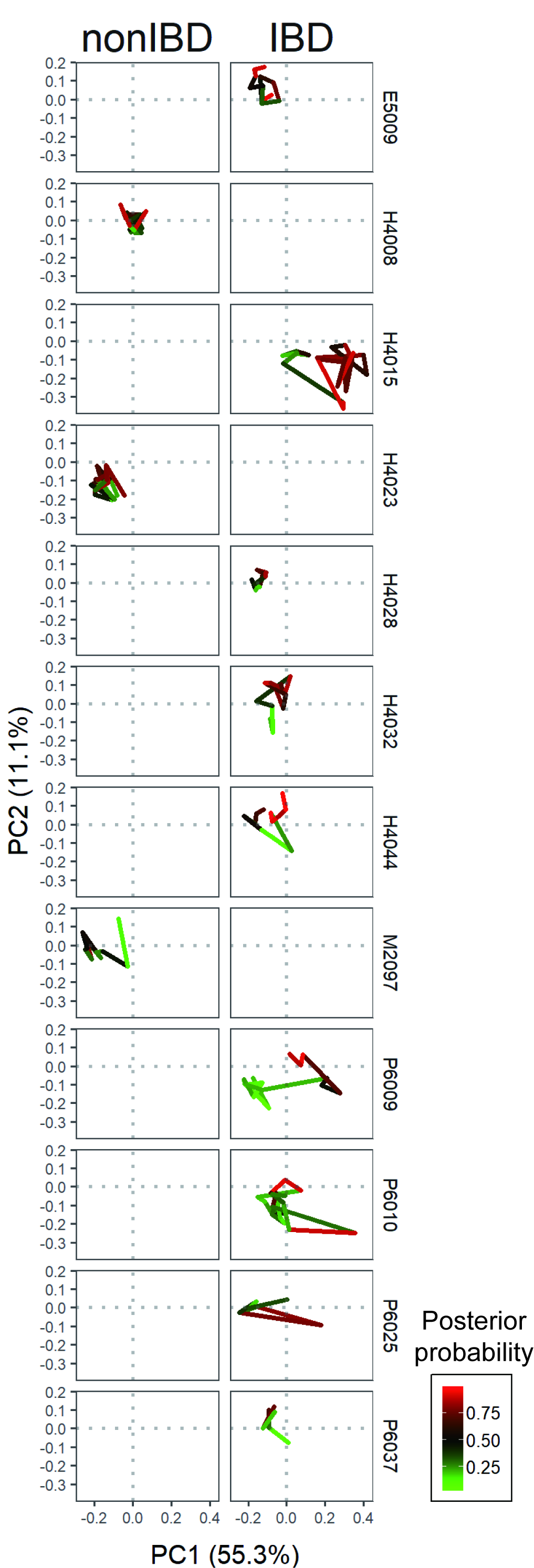

Supplement: Supplementary file 13 — Additional file 13. Figure S7. Individual alteration of microbiome in 12 dynamic subjects by inflammatory conditions and posterior probability. Line color indicates posterior probability. [file 12866_2020_1887_MOESM13_ESM.tif]

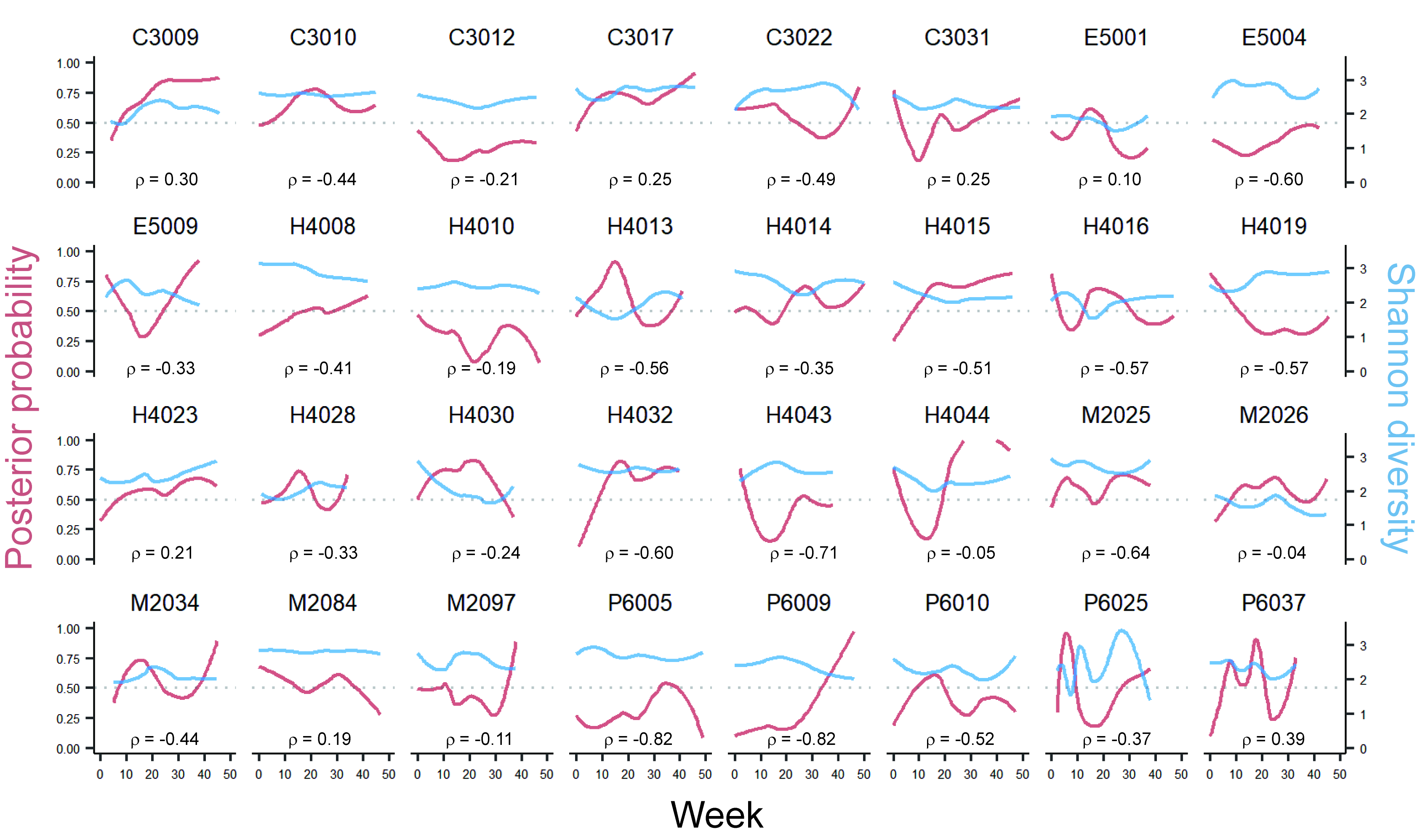

Supplement: Supplementary file 14 — Additional file 14. Figure S8. Intra-individual change of posterior probability and Shannon diversity in 70th percentile dynamic subjects. Pearson correlation coefficients were shown at the bottom of each participant panel. [file 12866_2020_1887_MOESM14_ESM.tif]

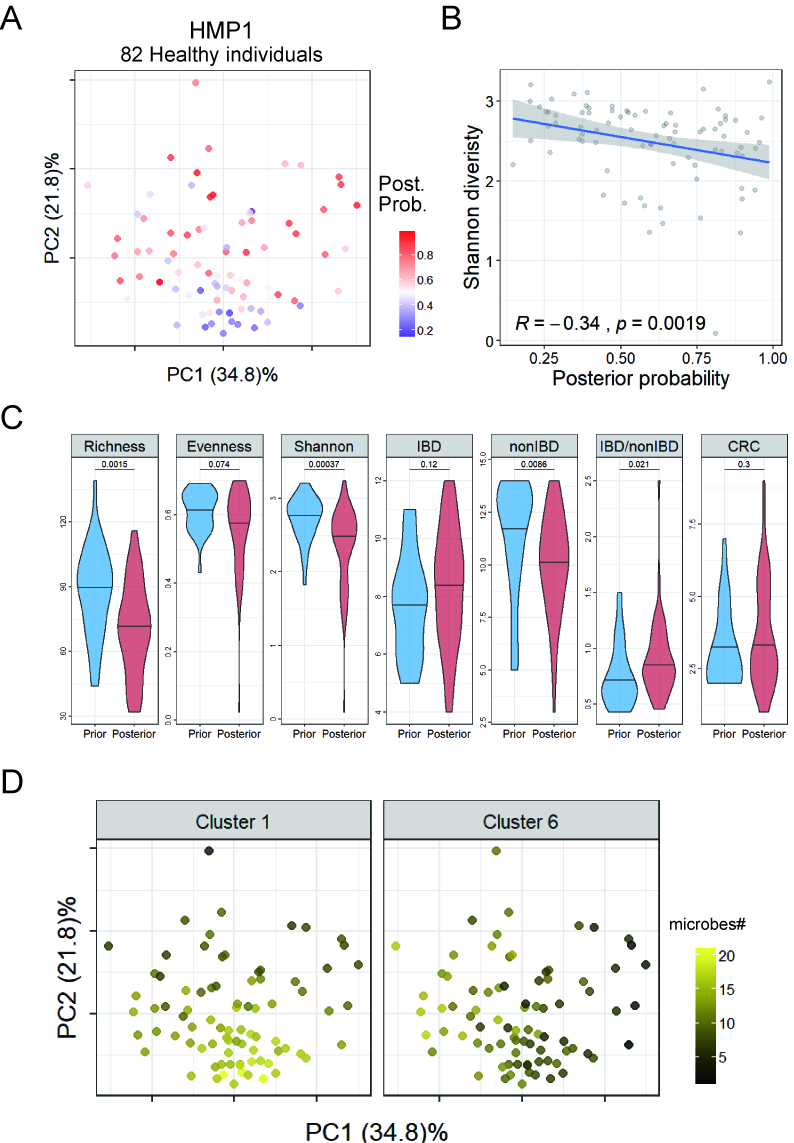

Supplement: Supplementary file 15 — Additional file 15. Figure S9. Model validation on independent healthy individuals. (a) Posterior probability of 82 fecal samples from healthy individuals. (b) Spearman correlation between posterior probability of F. nucleatum and Shannon diversity. (c) Microbial manifestations in putative F. nucleatum-prior or posterior samples. Wilcoxon rank sum test was performed. (d) prevalence of cluster 1 and 6 in validation dataset. * indicates p-value < 0.05, ** p < 0.011, *** p < 0.001. [file 12866_2020_1887_MOESM15_ESM.tif]
