# Supplementary material for: Opportunistic detection of Fusobacterium nucleatum as a marker for the early gut microbial dysbiosis
Source: BMC Microbiol. 2020 Jul 13;20:208. doi: 10.1186/s12866-020-01887-4 (PMC7359021; doi:10.1186/s12866-020-01887-4)
Supplement: Supplementary file 10 — Additional file 10. Table S5. Classifier species enriched prior or posterior to the detection point of F. nucleatum. [file 12866_2020_1887_MOESM10_ESM.docx]

Table 2. Classifier species that discriminate *F. nucleatum* observation point.

| microbe | Mean AUC | Std AUC | p-value (AUC>0.5) | FDR | NOD (out of 317) |
| --- | --- | --- | --- | --- | --- |
| *Dorea longicatena^$^* | 0.7224 | 0.0187 | 9.08E-33 | 2.34E-30 | 182 |
| *Coprococcus comes^$^* | 0.7143 | 0.0188 | 1.95E-30 | 5.03E-28 | 146 |
| *Lachnospiraceae bacterium 3_1_46FAA^$^* | 0.6924 | 0.0197 | 9.62E-23 | 2.48E-20 | 169 |
| *Roseburia hominis^$^* | 0.6594 | 0.0202 | 1.65E-15 | 4.25E-13 | 167 |
| *Roseburia inulinivorans^$^* | 0.6576 | 0.0204 | 5.18E-15 | 1.34E-12 | 183 |
| *Clostridium symbiosum^$^* | 0.6575 | 0.0195 | 2.96E-16 | 7.63E-14 | 205 |
| *Clostridium bolteae^$^* | 0.6427 | 0.0207 | 2.64E-12 | 6.80E-10 | 224 |
| *Bacteroides stercoris^$^* | 0.6421 | 0.0206 | 2.37E-12 | 6.12E-10 | 201 |
| *Alistipes shahii^$^* | 0.6400 | 0.0193 | 2.28E-13 | 5.89E-11 | 141 |
| *Veillonella parvula^$^* | 0.6356 | 0.0203 | 1.07E-11 | 2.76E-09 | 205 |
| *Collinsella aerofaciens^$^* | 0.6263 | 0.0203 | 2.65E-10 | 6.83E-08 | 206 |
| *Roseburia intestinalis* | 0.6140 | 0.0218 | 8.24E-08 | 2.13E-05 | 204 |
| *Bacteroides ovatus* | 0.6105 | 0.0204 | 3.17E-08 | 8.17E-06 | 240 |
| *Clostridium clostridioforme^$^* | 0.6102 | 0.0175 | 1.67E-10 | 4.30E-08 | 124 |
| *Bacteroides thetaiotaomicron* | 0.6058 | 0.0205 | 1.29E-07 | 3.33E-05 | 154 |
| *Clostridium citroniae^$^* | 0.6054 | 0.0165 | 9.25E-11 | 2.39E-08 | 97 |
| *Fusobacterium nucleatum* | 0.6044 | 0.0094 | 5.09E-29 | 1.31E-26 | 41 |
| *Flavonifractor plautii* | 0.6042 | 0.0202 | 1.18E-07 | 3.05E-05 | 225 |
| *Clostridium asparagiforme^$^* | 0.6036 | 0.0170 | 5.84E-10 | 1.51E-07 | 110 |
| *Burkholderiales bacterium 1_1_47* | 0.5986 | 0.0202 | 5.10E-07 | 0.000132 | 148 |
| *Faecalibacterium prausnitzii* | 0.5954 | 0.0201 | 1.09E-06 | 0.000282 | 289 |
| *Bifidobacterium bifidum* | 0.5950 | 0.0197 | 7.10E-07 | 0.000183 | 166 |
| *Bacteroidales bacterium ph8* | 0.5849 | 0.0129 | 2.74E-11 | 7.08E-09 | 60 |
| *Coprobacillus unclassified* | 0.5770 | 0.0167 | 1.97E-06 | 0.000509 | 96 |
| *Eubacterium sp_3_1_31* | 0.5597 | 0.0089 | 8.44E-12 | 2.18E-09 | 29 |
| *Proteus mirabilis* | 0.5593 | 0.0103 | 4.99E-09 | 1.29E-06 | 34 |
| *Desulfovibrio piger* | 0.5575 | 0.0082 | 1.20E-12 | 3.08E-10 | 25 |
| *Clostridium ramosum* | 0.5574 | 0.0124 | 1.90E-06 | 0.00049 | 50 |
| *Erysipelotrichaceae bacterium 5_2_54FAA* | 0.5544 | 0.0078 | 1.59E-12 | 4.10E-10 | 24 |
| *Parabacteroides johnsonii* | 0.5491 | 0.0081 | 8.38E-10 | 2.16E-07 | 22 |
| *Bacteroides sp_2_1_22* | 0.5479 | 0.0084 | 5.87E-09 | 1.52E-06 | 24 |
| *Morganella morganii* | 0.5434 | 0.0075 | 3.20E-09 | 8.25E-07 | 20 |
| *Peptostreptococcus stomatis* | 0.5406 | 0.0067 | 5.80E-10 | 1.50E-07 | 16 |
| *Collinsella intestinalis* | 0.5330 | 0.0058 | 7.79E-09 | 2.01E-06 | 13 |
| *Porphyromonas bennonis* | 0.5316 | 0.0066 | 6.79E-07 | 0.000175 | 15 |
| *Dysgonomonas unclassified* | 0.5306 | 0.0056 | 2.58E-08 | 6.65E-06 | 12 |
| *Megamonas hypermegale* | 0.5289 | 0.0064 | 2.96E-06 | 0.000763 | 14 |
| *Megamonas rupellensis* | 0.5288 | 0.0064 | 3.23E-06 | 0.000833 | 14 |
| *Dysgonomonas gadei* | 0.5256 | 0.0051 | 3.09E-07 | 7.98E-05 | 10 |
| *Anaerofustis stercorihominis* | 0.5255 | 0.0051 | 3.36E-07 | 8.67E-05 | 10 |
| *Proteus unclassified* | 0.5253 | 0.0050 | 2.35E-07 | 6.07E-05 | 10 |

* Statistic significance for each microbe to discriminate *F. nucleatum*-posterior samples from *F. nucleatum*-prior ones was iteratively calculated from 100 random dataset. Classifier species were filtered by FDR (<0.001) and ordered by mean AUC.

* $ indicates potent classifiers having FDR < 1e-07 and average AUC > 0.6. They were selected as features for logistic regression modeling.

* NOD indicates the detection frequency of microbes out of 317 samples from *F. nucleatum*-experienced subjects.

* See methods for detailed information.
