# Supplementary material for: Opportunistic detection of Fusobacterium nucleatum as a marker for the early gut microbial dysbiosis
Source: BMC Microbiol. 2020 Jul 13;20:208. doi: 10.1186/s12866-020-01887-4 (PMC7359021; doi:10.1186/s12866-020-01887-4)
Supplement: Supplementary file 11 — Additional file 11. Table S6. Correlation coefficients with F. nucleatum and multiple enrichment tests for global biomarker species of colorectal cancer (CRC). [file 12866_2020_1887_MOESM11_ESM.docx]

| microbe | Spearman ρ  (P-value) | Non-IBD | IBD | *F. nucleatum*-  innocent | *F. nucleatum* -experienced | *F. nucleatum* -prior | *F. nucleatum* -posterior | NOD^$^  (%) |
| --- | --- | --- | --- | --- | --- | --- | --- | --- |
| *Anaerococcus*  *obesiensis* | 0.0821  (0.0013) | 0.9776 | 0.0224 | 0.4137 | 0.5866 | 0.8665 | 0.1355 | 11  (0.72) |
| *Anaerococcus*  *vaginalis* | 0.1221  (1.72E-06) | 0.5557 | 0.4447 | 0.6393 | 0.3610 | 0.9131 | 0.0881 | 12  (0.79) |
| *Clostridium*  *bolteae* | 0.1100  (1.65E-05) | 1 | 4.36E-19 | 0.9195 | 0.0806 | 1.0000 | 7.02E-06 | 1030  (67.50) |
| *Clostridium*  *clostridioforme* | 0.0940  (0.0002) | 1.0000 | 6.07E-09 | 0.9980 | 0.0020 | 1.0000 | 9.65E-05 | 459  (30.08) |
| *Clostridium*  *symbiosum* | 0.0733  (0.0042) | 1 | 8.58E-17 | 1 | 1.75E-13 | 1.0000 | 8.14E-07 | 731  (47.90) |
| *Fusobacterium*  *nucleatum* | NA | 0.9242 | 0.0758 | 1 | 4.32E-37 | 1.0000 | 4.93E-08 | 41  (2.69) |
| *Gemella*  *morbillorum* | 0.2052  (4.44E-16) | 0.9116 | 0.0886 | 0.9849 | 0.0152 | 0.9131 | 0.0881 | 5  (0.33) |
| *Peptostreptococcus*  *stomatis* | 0.3210  (0) | 0.9950 | 0.0050 | 1 | 4.15E-13 | 0.9993 | 0.0007 | 18  (1.18) |
| *Parvimonas*  *micra* | 0.1699  (2.40E-11) | 0.9451 | 0.0550 | 0.9911 | 0.0089 | 0.9420 | 0.0588 | 7  (0.46) |
| *Parvimonas*  *unclassified* | 0.1763  (4.03E-12) | 0.9938 | 0.0062 | 0.9995 | 0.0005 | 0.9550 | 0.0455 | 17  (1.11) |
| *Porphyromonas*  *asaccharolytica* | 0.0916  (0.0003) | 0.0951 | 0.9050 | 0.9713 | 0.0287 | 0.9014 | 0.0991 | 115  (7.45) |
| *Porphyromonas*  *somerae* | 0.1408  (3.31E-08) | 0.0002 | 0.9998 | 0.9998 | 0.0002 | 0.8365 | 0.1645 | 24  (1.57) |
| *Porphyromonas*  *uenonis* | 0.0886  (0.0005) | 0.2782 | 0.7220 | 0.9487 | 0.0514 | 0.9039 | 0.0967 | 46  (3.01) |
| *Prevotella*  *intermedia* | -0.0043  (0.8681) | 0.7273 | 0.2737 | 0.3049 | 0.6963 | 1 | 1 | 1  (0.07) |
| *Prevotella*  *nigrescens* | -0.0074  (0.7735) | 0.8522 | 0.1481 | 0.7039 | 0.2967 | 0.1012 | 0.9011 | 3  (0.20) |
| *Ruminococcus*  *torques* | 0.05396  (0.0351) | 0.3303 | 0.6697 | 0.9579 | 0.0421 | 0.1063 | 0.8940 | 1402  (91.87) |
| *Solobacterium*  *moorei* | 0.1703  (2.12E-11) | 0.9820 | 0.0180 | 0.8611 | 0.1391 | 0.9420 | 0.0588 | 12  (0.79) |
| *Subdoligranulum*  *unclassified* | -0.1041  (4.59E-05) | 2.69E-24 | 1 | 1.72E-13 | 1 | 0.0088 | 0.9912 | 1464  (95.94) |

Table 3. Correlation coefficients with F. nucleatum and multiple enrichment significance of CRC biomarker species.

* All tests were Wilcoxon rank-sum test. $ is the number of detection of each microbe across 1526 samples.
